# Supplementary material for: Best-practice guidance for Earth BioGenome Project sample collection and processing: progress and challenges in biodiverse reference genome creation
Source: Gigascience. 2025 May 29;14:giaf041. doi: 10.1093/gigascience/giaf041 (PMC12121479; doi:10.1093/gigascience/giaf041)
Supplement: giaf041_GIGA-D-24-00457_Original_submission [file giaf041_giga-d-24-00457_original_submission.pdf]

GigaScience

Best Practice Guidance for Earth BioGenome Project Sample Collection and Processing: Progress and Challenges in Biodiverse Reference Genome Creation

--Manuscript Draft--

**Manuscript Number:**	GIGA-D-24-00457
**Full Title:**	Best Practice Guidance for Earth BioGenome Project Sample Collection and Processing: Progress and Challenges in Biodiverse Reference Genome Creation
**Article Type:**	Review
**Funding Information:**	Wellcome Trust (220540/Z/20/A) — Dr Mark Blaxter
**Abstract:**	The Earth BioGenome Project (EBP) has the extremely ambitious goal of generating, at scale, high quality reference genomes across the entire Tree of Life [1]. Currently in its first phase, the project is targeting family-level representatives and is progressing rapidly. Here we outline recommended standards and considerations in sample acquisition and processing for those involved in biodiverse reference genome creation. As a living document, these standards and recommendations will evolve with advances in related processes. Additionally, we discuss the challenges raised by the ambitions for later phases of the project, highlighting topics related to sample collection and processing that require further development.
**Corresponding Author:**	Mara Lawniczak<br>Wellcome Sanger Institute<br>Hinxton, UNITED KINGDOM
**Corresponding Author Secondary Information:**	
**Corresponding Author's Institution:**	Wellcome Sanger Institute
**Corresponding Author's Secondary Institution:**	
**First Author:**	Mara Lawniczak
**First Author Secondary Information:**	
**Order of Authors:**	Mara Lawniczak
	Kevin Kocot
	Jonas Astrin
	Mark Blaxter
	Cibele Sotero-Caio
	Katharine Barker
	Anna Childers
	Jonathan Coddington
	Paul Davis
	Kerstin Howe
	Warren Johnson
	Duane McKenna
	Jeremy Wideman
	Bernardo Santos
**Order of Authors Secondary Information:**	
**Additional Information:**	

Powered by Editorial Manager® and ProduXion Manager® from Aries Systems Corporation

Question	Response
Are you submitting this manuscript to a special series or article collection?	No
**Experimental design and statistics**<br><br>Full details of the experimental design and statistical methods used should be given in the Methods section, as detailed in our Minimum Standards Reporting Checklist. Information essential to interpreting the data presented should be made available in the figure legends.<br><br>Have you included all the information requested in your manuscript?	No
If not, please give reasons for any omissions below.<br><br>as follow-up to "**Experimental design and statistics**<br><br>Full details of the experimental design and statistical methods used should be given in the Methods section, as detailed in our Minimum Standards Reporting Checklist. Information essential to interpreting the data presented should be made available in the figure legends.<br><br>Have you included all the information requested in your manuscript?<br><br>"	N/A
**Resources**<br><br>A description of all resources used, including antibodies, cell lines, animals and software tools, with enough information to allow them to be uniquely identified, should be included in the Methods section. Authors are strongly encouraged to cite Research Resource Identifiers (RRIDs) for antibodies, model	No

Powered by Editorial Manager® and ProduXion Manager® from Aries Systems Corporation

organisms and tools, where possible.<br><br>Have you included the information requested as detailed in our Minimum Standards Reporting Checklist?	
If not, please give reasons for any omissions below.<br><br>as follow-up to "**Resources**<br><br>A description of all resources used, including antibodies, cell lines, animals and software tools, with enough information to allow them to be uniquely identified, should be included in the Methods section. Authors are strongly encouraged to cite Research Resource Identifiers (RRIDs) for antibodies, model organisms and tools, where possible.<br><br>Have you included the information requested as detailed in our Minimum Standards Reporting Checklist?<br><br>"	N/A
**Availability of data and materials**<br><br>All datasets and code on which the conclusions of the paper rely must be either included in your submission or deposited in publicly available repositories (where available and ethically appropriate), referencing such data using a unique identifier in the references and in the "Availability of Data and Materials" section of your manuscript.<br><br>Have you have met the above requirement as detailed in our Minimum Standards Reporting Checklist?	No
If not, please give reasons for any omissions below.	N/A

as follow-up to "**Availability of data and materials**<br><br>All datasets and code on which the conclusions of the paper rely must be either included in your submission or deposited in publicly available repositories (where available and ethically appropriate), referencing such data using a unique identifier in the references and in the "Availability of Data and Materials" section of your manuscript.<br><br>Have you have met the above requirement as detailed in our Minimum Standards Reporting Checklist?<br><br>"	
GigaScience has policies and guidelines in place for the use of generative AI-writing tools such as ChatGPT. If you have used such writing tools to assist with writing the manuscript this must be declared and cited in the text. Authors should not list AI-writing tools and other AI-assisted technologies as an author or co-author and should acknowledge that they are fully responsible for text generated or refined by AI-writing tools.<p><br><br>A summary of use (particularly in the introduction or among methods) needs to be included at the end of the paper, and the outputs should also be included as a supplementary file hosted in GigaDB or other open repositories. Please read our guidelines for more information. <p><br><br>By submitting to GigaScience, you are aware of the journal's AI-writing tools policy, and if you have declared use of such tools below, you have acknowledged this where appropriate in your manuscript and have made a summary of use and outputs available. <p><br><b>AI-assisted writing tools have been used in the preparation of this manuscript?	No

Click here to access/download;Manuscript;EBP Sample Collection and Processing Best Practices.pdf

Best Practice Guidance for Earth BioGenome Project Sample Collection and Processing: Progress and Challenges in Biodiverse Reference Genome Creation

Mara K. N. Lawniczak[1], Kevin M. Kocot[2,], Jonas J. Astrin[3], Mark Blaxter[1], Cibele G. Sotero-Caio[1], Katharine B. Barker[4], Anna K. Childers[5], Jonathan Coddington[4], Paul Davis[1], Kerstin Howe[1], Warren E. Johnson[4], Duane D. McKenna[6,7], Jeremy Wideman[8], Olga Vinnere Pettersson[9], Verena Ras[10,11], Bernardo F. Santos[12], and the Earth BioGenome Project Samples and Processing sub-Committee[13]

1. Tree of Life, Wellcome Sanger Institute, Hinxton, CB10 1SA United Kingdom
2. Department of Biological Sciences & Alabama Museum of Natural History, University of Alabama, Tuscaloosa, AL, USA
3. Leibniz Institute for the Analysis of Biodiversity Change, Museum Koenig, Bonn, Germany
4. National Museum of Natural History, Smithsonian Institution, Washington, DC, USA
5. USDA, Agricultural Research Service, Beltsville Agricultural Research Center, Bee Research Laboratory, 10300 Baltimore Avenue, Beltsville, MD 20705, USA
6. Department of Biological Sciences, University of Memphis, Memphis, TN 38152, USA
7. Center for Biodiversity Research, University of Memphis, Memphis, TN 38152, USA
8. Biodesign Center for Mechanisms of Evolution, School of Life Sciences, Arizona State University, Tempe, AZ, 85287, USA
9. SciLifeLab, National Genomics Infrastructure, Department of Immunology, Genetics and Pathology, Uppsala University, Uppsala, SE-751 05, Sweden
10. Computational Biology Division, Department of Integrative Biomedical Sciences, University of Cape Town, Cape Town, South Africa.
11. Department of Biodiversity and Conservation Biology, University of the Western Cape, Cape Town, South Africa.
12. Museum für Naturkunde, Leibniz Institute for Evolution and Biodiversity Science, Center for Integrative Biodiversity Discovery, Invalidenstraße 43, Berlin 10115, Germany
13. https://www.earthbiogenome.org/sample-collection-processing

ABSTRACT

The Earth BioGenome Project (EBP) has the extremely ambitious goal of generating, at scale, high quality reference genomes across the entire Tree of Life [1]. Currently in its first phase, the project is targeting family-level representatives and is progressing rapidly. Here we outline recommended standards and considerations in sample acquisition and processing for those involved in biodiverse reference genome creation. As a living document, these standards and recommendations will evolve with advances in related processes. Additionally, we discuss the challenges raised by the ambitions for later phases of the project, highlighting topics related to sample collection and processing that require further development.

BACKGROUND

The Earth BioGenome Project (EBP) comprises a network of local, continental, and taxon focused projects (https://www.earthbiogenome.org/affiliated-project-networks), all of which are contributing to the vision that reference genomes across the Tree of Life have and will have huge impact on our understanding of the world around us and on planetary health. The EBP is currently in its first phase, with many projects already contributing and many more projects that may be initiated in the near future. EBP Phase I proposes the sequencing of a representative species for all of the ~9,500 described eukaryotic families (see https://www.catalogueoflife.org/). For the majority of Phyla, a genome is available but many of these do not reach chromosomal level (Figure 1). With respect to the Phase I goal, as of October 2024, there are genomes for 3000 species from over 1000 families, of which over 2000 species from over 750 families have chromosome level assemblies. Thus, significant work remains to complete Phase I. This document aims to provide recommendations for any project that is generating reference genomes.

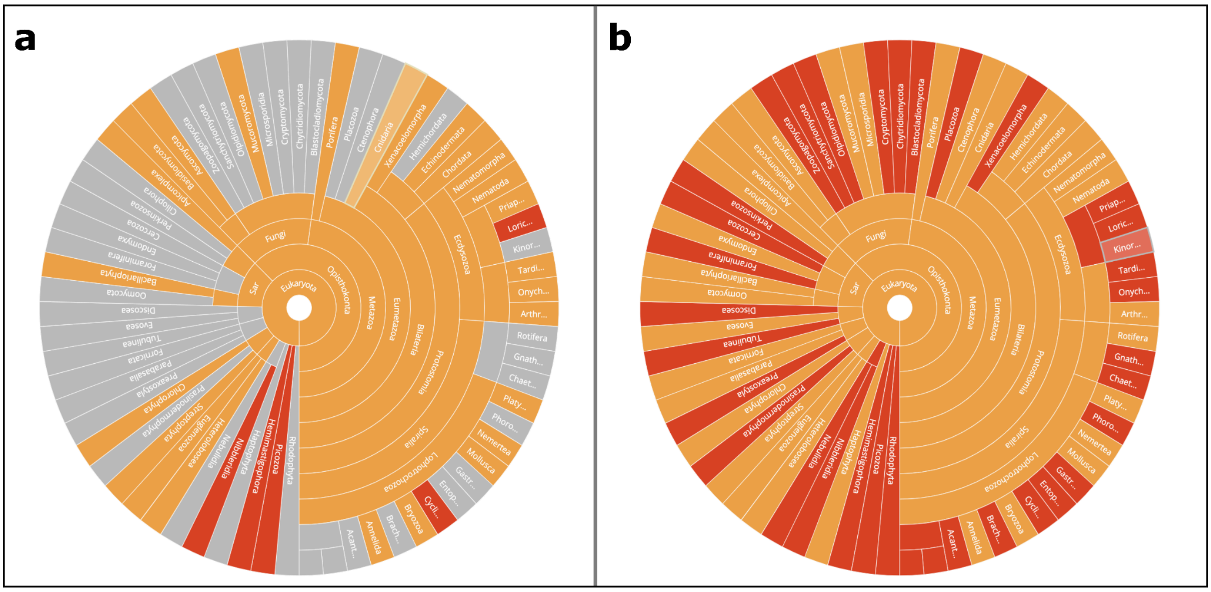

Figure 1. Genome sequencing across Eukaryotic phyla from NCBI taxonomy. On the left, all assembly levels: red- no available genomes; orange - at least 1 descendent sequenced by the EBP; grey - at least 1 descendent has an available genome. GoaT link for interactive tree. On the right, in orange are phyla with at least 1 EBP-quality assembly and in red, those with no available EBP-quality genomes. GoaT link for interactive tree.

Recommendations for success in sampling for biodiversity genomics

Here, we list general factors to consider as projects set out to select their target species, and then we discuss at more length some additional considerations. While this list is focused on EBP Phase 1, most of the recommendations are advisable even for projects that already have a clear species target list. This list is focused on larger multicellular organisms or smaller species that are easily cultured, but we discuss below ultra-low input approaches for protists and other species where physical size is a limitation. General factors to consider when suggesting a species representative towards the EBP Phase I goal include:

- **Community value**: Species should be of broad community use and value including species that are of economic or ecological value, are of conservation concern, have specific scientific interest, or have iconic status. Community value could be assessed through surveys oriented towards target communities (e.g.: https://tinyurl.com/dtol-suggest for the Darwin Tree of Life project).
- **Permissions and availability**: Sampling should be achievable, considering permissions and ethical and legal collection requirements, as well as ease of collection (geography, collecting method).
- **Publicly registered:** The species should be registered with its current name and taxonomy in a publicly available database (we recommend the NCBI Taxonomy Database https://www.ncbi.nlm.nih.gov/taxonomy) and assigned a numeric identifier to assist with tracking taxonomic changes over time. This is covered in more detail below.
- **Taxonomic stability & representation**: The taxon should not be subject to current disagreement and revision. From a taxonomic perspective, it is preferable to sample the type genus of the family, or at least a taxon from the type genus subfamily. The species should be generally considered a “good” biological species (not from a known species complex) and if possible, sampled from or near the type locality.
- **Voucher availability:** If the organism is large, multiple subsamples should be taken for tissue and nucleic acid biobanking. For smaller organisms that are likely to be consumed in genome generation, ideally several additional individuals from the same time and place would be collected for morphological and molecular vouchering (e.g. biobanking of DNA, RNA, and/or tissue). Likewise, photographic documentation of living specimens should be undertaken whenever possible.

Additional factors to consider for multicellular organisms include:

- **Genome size, ploidy, sex, and life stage(s)**: Where genome sizes and/or ploidy are known (estimates are available via Genomes on a Tree (GoaT); see below), prioritize species with smaller, diploid genomes. This is because data generation costs will fall and our ability to assemble high repeat content genomes will improve in future EBP phases. Where sex chromosomes are understood, selecting the heterogametic sex if possible provides a more complete view of the genome. For species with haplodiploid sex determination, the haploid sex should be chosen.
- **Physical sample size**: We recommend a minimal requirement of ten samples, each weighing more than 10 milligrams per 1 Gb of genome size, for animals and multicellular / culturable fungi, culturable protists and 100 milligrams per 1 Gb of genome size for plants and multicellular algae. A minimum of three samples is required to support the three platforms of long read, chromatin conformation (Hi-C), and transcriptome (RNAseq) sequencing. Depending on genome size, amplification-based long-read sequencing approaches may still result in a high-quality reference genome for organisms where this is not possible.

The considerations listed above are not intended to be a “must have” list and each project will need to determine what is important for their particular aims. For example, one project might deem completing the genome of a single 9 Gb locust species to be too expensive when those funds could support genomes for 20 species, but another project might deem the locust genome extremely important. In other words, the prioritization of the considerations listed above should be made by each project independently.

In addition to the considerations listed above, there are a number of other areas key to successfully and ethically contributing to EBP goals. Here we set out considerations and recommendations in each of these areas.

EBP Phase I family-representative target species: openly collating community proposals and genomes underway

As the technology needed to generate high quality reference genomes is improving and becoming increasingly globally accessible, global coordination to avoid duplication of effort (i.e., sequencing the same species) where possible is important. Multiple species for each

family should be proposed and collected to provide greater flexibility in achieving Phase I goals. As much as possible, this process should be globally visible, with species underway and their associated projects made clear. All projects should use the Genomes on a Tree (GoaT) system (https://goat.genomehubs.org/, [2]) to share their target lists and progress. GoaT has a searchable interface that not only lists a constantly updated database of progress reported from ongoing reference genome projects, but also provides direct and inferred values for genome size, ploidy, and chromosome number. These are extremely useful metrics when selecting target species.

In addition to assisting with global coordination, contributing lists of target species to GoaT allows genomes sequenced through these projects to be tracked and counted both to local and EBP Phase I goals. These target lists declare intent, and as such, are likely to undergo revision as projects proceed due to, for example, challenges with acquiring specific target species or extracting adequate DNA and RNA from them. We recommend that larger projects create and maintain a live file listing their target species, identifying taxa selected as EBP family representatives (Figure 2a), and supply the file URL to the GoaT data curators. The format for the file is given in https://goat.genomehubs.org/submissions, and a 2024 version is presented at http://tinyurl.com/goat-submission-template. GoaT archives the submitted file and processes it for display. Larger funded projects are often open to receiving suggestions for target taxa and we encourage genome users to request particular species from the most relevant existing EBP projects (e.g. for an African species, contact AfricaBP).

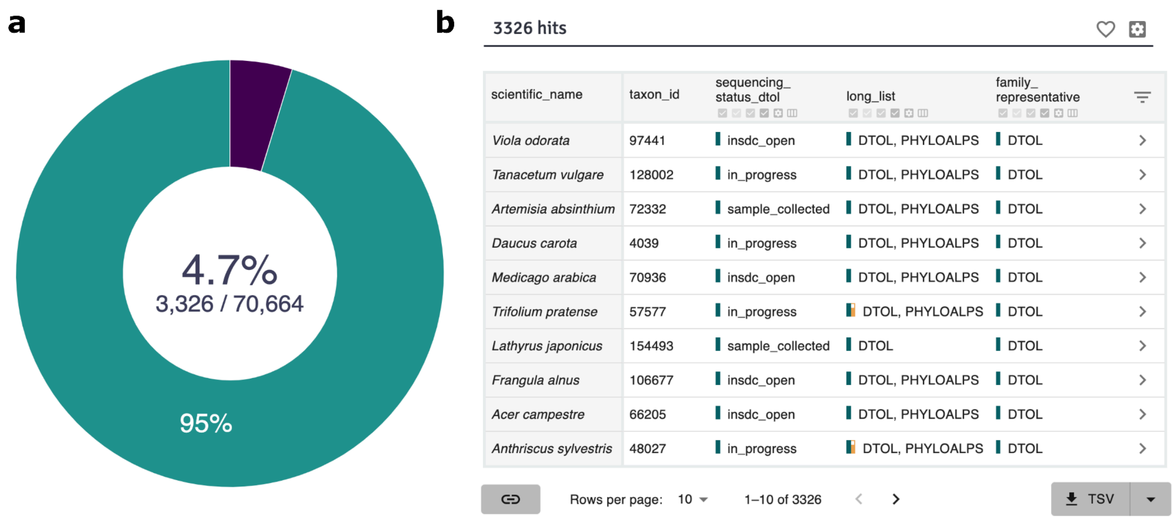

Figure 2. Example of a search for family representatives on GoaT. a. Report of Darwin Tree of Life (DToL) species proposed as family representatives out of all >60K species on the DToL wishlist. b. List of species from the report in (a.) with additional columns displaying target (scope) overlaps when present (long_list = DToL, PHYLOALPS) and current sequencing status within the DToL genome production pipeline. GoaT search link.

GoaT can import lists of intent and progress for smaller projects if the project is registered under an umbrella bioproject ID on INSDC and ideally linked to at least one major EBP initiative. Projects should contact one of the major initiatives that best fit their scope to be listed as a contributing lab. The main contact for each project can be found in each project-dedicated page at https://goat.genomehubs.org/projects. Target lists and contact information on GoaT are public and resolution of any overlaps (e.g., Figure 2b) can be initiated by either project via the contact information provided.

Use cases for exploring available EBP data and also identifying sequencing gaps in taxonomic groups can be found at https://goat.genomehubs.org/help/use_cases. Summary information and live progress reports are available as a project-dedicated page for all EBP affiliates at https://goat.genomehubs.org/projects. Each project page contains duplication checkers, where the overlap of target species or progress can be cross-referenced among EBP affiliates. Projects should take advantage of the duplication checkers on GoaT to negotiate and split tasks to maximize the number of species sequenced, and to remove species from target lists by filtering to show those meeting EBP reference genome standards (https://www.earthbiogenome.org/report-on-assembly-standards).

Ethical Collecting

It is paramount that specimens and projects contributing to the EBP are both legally and ethically obtained. Sample collectors should ensure that all local and national permissions for collection are in place, and that there is a record of these permissions that can be referred to if any questions arise as to whether a specimen was legally obtained. This guidance applies to all species, not just those that are of conservation concern. These permissions will vary widely between countries and jurisdictions within them (e.g. [3,4] for ERGA, European Reference Genome Atlas), so it is beyond the scope of this document to summarize them. Best practice is to ensure that every specimen is collected legally within the applicable frameworks including national and local rules, rules on endangered species, rules on collecting in protected sites, and rules regarding traditional knowledge. It is also important that permissions are obtained for sequencing, shipping, and publication.

Another complicating factor is that at this phase in the project, some specimens will likely be collected and moved out of their country of origin for sequencing. The Nagoya Protocol (http://www.cbd.int/cop10/) and/or Access and Benefit Sharing (ABS) policies must be followed in these cases. Precise guidance on following the Nagoya Protocol is beyond the scope of this document, especially given that many countries interpret the protocol differently. At a broad level, sample collectors who will be shipping specimens outside their country of origin must contact their local ABS Clearing-house (https://www.cbd.int/abs/) to understand what the rules are, and to obtain a PIC (Prior Informed Consent) document and a MAT (Mutually Agreed Terms on what the benefit is: financial or academic, an acknowledgement on a paper, sharing results, etc). These documents should be written as broadly as possible to support the project's vision. Countries receiving samples should ensure they have further permissions within the MAT to pass the samples on if there is any anticipation that might be required, for example to support biobanking and morphological vouchering and how data may be shared. Further considerations from the EBP's Ethical, Legal, and Social Issues (ELSI) committee can be found in Sherkow et al. 2022 [5]

Beyond the rules and regulations, collecting methods must be ethical. Effort should be made to build sustainable partnerships with indigenous peoples and local communities [3]. Overcollection of any species should be avoided. Projects should consider what the best

sampling strategies might be to avoid overcollection, for example, lineage-focused bioblitzes with a group of taxonomic experts.

Confident Species Identification

Each contributed specimen should be identified to species level by a taxonomic expert and ideally, material from the same specimen should be independently DNA barcoded using appropriate markers and the data deposited publicly on BOLD (http://www.boldsystems.org) or in an INSDC database. These DNA barcodes will ensure that species with reference genomes have independently generated DNA barcode data, that the DNA barcodes match the resulting reference genome, and that no sample swaps occur along the way.

Robust and Complete Metadata

Robust and complete metadata of all types must accompany the family-level representatives for EBP. Metadata fields and terms should be standardized and we recommend drawing on the extensive efforts to standardize collection metadata already completed by the Biodiversity Information Standards community (originally the Taxonomic Databases Working Group, TDWG; see https://www.tdwg.org/) including the Darwin Core standards (https://www.tdwg.org/standards/dwc/), Access to Biological Collection Data (ABCD) Schema, https://www.tdwg.org/standards/abcd/, and GGBN Data Standard, an extension of the above for molecular samples (https://www.tdwg.org/standards/ggbn/). The latest TDWG guidance can be found at https://www.tdwg.org/standards/. The Darwin Tree of Life (DToL) project has adapted these for collections for biodiversity genomics [6], and the latest DToL guidance can be found at https://github.com/darwintreeoflife/metadata/tree/main. Other projects have already built on the foundation provided by DToL (e.g., https://github.com/ERGA-consortium/ERGA-sample-manifest) and it is important to maintain the ontology of terms used in these different projects to prevent drift whereby terms become project-specific. Therefore it is advisable to use these metadata schemas as they are or to ensure that modifications are made in consultation. If project-specific metadata differ substantially from these EBP recommendations, other INSDC sample checklists (e.g., https://www.ebi.ac.uk/ena/browser/checklists or

https://www.ncbi.nlm.nih.gov/biosample/docs/packages/) are perfectly acceptable, provided that as many metadata fields are completed as is possible and reasonable.

The EBP recommends that every specimen should have a ToLID (tree of life identification) assigned. A ToLID is a unique, easy to communicate identifier that provides species recognition, numerically differentiates between specimens of the same species, and adds some taxonomic context. ToLIDs facilitate internal and external communication about the samples and help the EBP track all sequencing projects. It is also worth remembering that a specimen can contain multiple organisms so ToLID can disambiguate between target and off target organisms within a given sample/specimen allowing other specimen products to be published unambiguously. Further, when an assembly is submitted, the ToLID should be used to name the assembly in order to provide unambiguous tracking of which samples were used for specific data types. When more than one specimen is used to generate the multiple data types necessary for an assembly, current guidance is to name the assembly using the ToLID that was used for long read data generation. ToLIDs are not a replacement for INSDC BioSample records, which hold all of the metadata associated with the sample. Every sample should have both. ToLIDs can be requested at https://id.tol.sanger.ac.uk/ (instructions on the website).

To facilitate tracking of sequencing status at the species level, and retrieving biodiversity genomics data via stable taxon identifiers, the EBP recommends assigning taxon identifiers (taxids) in the NCBI Taxonomy as early as possible in the creation of a draft target list. Taxids are necessary not only for registering a ToLID but also for declaration of intent on GoaT and for generation of BioSamples and assembly submissions. Specific guidelines have been created [7] to identify the need to-, what type of- and how to- request taxids for planned sequencing targets. The main steps include checking availability of a taxid for the target species using ENA or NCBI Taxonomy query services, using NCBI and ENA web interfaces, along with other taxonomy-dedicated sources to identify potential synonyms and names that need reconciliation across sources, requesting from ENA or NCBI a new taxid for species (or if needed, subspecies) not yet in NCBI Taxonomy, and for species-like entries for which the full Linnean binomen is not determined. Researchers are encouraged to discuss directly with the NCBI Taxonomy curators (or the curators at ENA)whenever there is an opportunity to improve the NCBI Taxonomy, including taxonomic revisions and reconciliations.

It is important to develop a robust mechanism for updating the names and identifiers associated with a specimen if misidentification or taxonomic reclassification occurs while a reference genome is being produced. This mechanism should also have a policy to determine what gets changed at different points in the process. If a misidentification or a taxonomic name change occurs early in the process of data generation then fully updating to a new taxid, scientific name, and ToLID would be appropriate. Once the assembly has been published, we recommend correcting the taxonomic information and ToLID in any cases of misidentifications, even after release. However, taxonomic changes like synonym preferences or merges should only be cause for amendments within 6 months after assembly release and the ToLID should remain as is.

Vouchering

Voucher specimens from each species contributing to a reference genome should be preserved when possible and guidance on various biobanking topics is available [8,9]. Ideally, there will be both morphological vouchers preserving the diagnostic characters of the taxon (or image vouchers if no material sample can be preserved), and molecular vouchers, such as tissue vouchers (discussed below), viably frozen cell lines, and/or extracted RNA and DNA. Vouchers should be prepared in a manner that ensures long-term physical preservation of the specimen and straightforward association with its metadata. Vouchers should be deposited in publicly accessible collection facilities, such as natural history museums or herbaria, located in the country of origin for each sequenced species, or where there is excess material, possibly spread across multiple repositories. It is advisable to arrange in advance of the work commencing where remaining material (including vouchers) should reside after the work is completed, and this should be reflected in any relevant legal transfer agreements. It can otherwise require secondary legal agreements to transfer materials to a third party.

It is recommended that molecular vouchers be stored in a GGBN member institution for sample accessibility, and linked to the morphological voucher using a unique ID, ideally a globally unique ID (GUID, https://datatracker.ietf.org/doc/html/rfc4122), or at least following the Darwin Core triplet structure ([unique institute acronym][collection acronym, unique in the institute][voucher number, unique in the collection]). Any facility that owns and/or manages collections of non-human genomic/molecular samples can apply to become a GGBN member institute, by following the instructions here:

https://wiki.ggbn.org/ggbn/Membership. It is also worth bearing in mind that if you are sharing material with another institute and an MTA (material transfer agreement) is in place, make sure that the MTA states that remaining material can be shared with a third party if you so wish.

High-quality images should accompany each contributed specimen, and these should be made publicly available. Photographic documentation of specimens should include all relevant morphological axes. If the specimens were collected while actively engaging in identifiable behaviors (mating, feeding) these should be photographed and noted as well as the time of collection. These images can be deposited in the BioImage Archive (https://www.ebi.ac.uk/bioimage-archive/), GBIF (GBIF.org), or iDigBio (iDigBio.org). For genomes accompanied by a DNA barcode, the BOLD database may also be an option for image archiving, as well as GGBN if samples are biobanked in a member repository.

Sample to Sequence

The Phase I ambition for EBP is to sequence species that are good family-level representatives and the guidelines above indicate the primary considerations for selecting appropriate species. Ultimately, what is selected and sequenced is an individual or a set of individuals and should be recognized as so. Furthermore, additional considerations when selecting the precise specimen(s) that will be sequenced to represent the species and the family are discussed below.

Specimen and Tissue Selection

The sex of the specimen and the particular life history stages and tissues that are best for different data types should be considered carefully. Where relevant and possible, it is preferable to sample from the heterogametic sex to provide data for both sex chromosomes. Recommendations on the best life stages and best tissues to target to achieve the highest qualities and quantities of DNA, RNA, and nuclei will vary depending on the taxon. When possible, if a species has been described from a given life stage, that life stage should be selected for sequencing to decrease the likelihood of misidentification. Specific tissues might be prioritized or avoided based on additional species (cobionts) that might be present in or associated with those tissue types (e.g., sequencing gut tissue may yield off-target sequences that may or may not be desirable). The exact tissue types

recommended for High Molecular Weight (HMW) DNA and RNA for the wide range of target taxa are beyond the scope of this document. They undoubtedly will change as we experience successes and failures in extracting nucleic acids, preparing libraries, sequencing, assembly and annotation. For annotation using RNAseq, see the EBP guidelines (https://www.earthbiogenome.org/report-on-annotation-standards). Briefly, it is recommended to collect a diversity of tissue types whenever possible, factoring in previous understanding of tissues for the focal taxa that have representative or higher-than-average transcript diversity. Over time, we will gain a better understanding of the life stages and tissues for a wide range of taxa that provide the best quantities and qualities of the material along with the most reliable protocols (e.g. for RNA extraction and for sequencing).

The target individual should be collected from the wild rather than from a laboratory colony, zoo, or culture collection (when possible), to capture natural genetic diversity, avoid inbreeding effects, and accurately reflect ecological and evolutionary contexts [10]. The size of the samples needed from a specimen will depend on the taxon and tissue sampled and the genome size, and the precise guidance around required input material is likely to be a rapidly moving target as required quantities decrease and our ability to achieve high-quality extracts across a wide range of taxa increases. Our current recommendations for animals and multicellular fungi are at least 10 and preferably closer to 100 milligrams of tissue per 1 Gb of genome size for each sample as this tends to be sufficient for long-read data generation without amplification. For plants and multicellular algae we suggest at least 100 and preferably 1000 milligrams of tissue per Gb of genome for each sample. Multiple samples from the same specimen should be prioritized over single samples from different specimens, and given the current need for samples to be directed down three different processes (Hi-C, HMW DNA extraction, RNA extraction), we recommend at least 10 samples meeting these standards per species. For taxa that are known to be difficult (e.g., many marine invertebrates) and might require many extraction attempts, at least 20 samples should be taken given the significant delays and costs incurred by the need for an additional collection trip. If taking a sufficient number of samples from a single specimen is not possible, then additional specimens, ideally from the same locality and collected at the same time, should be preserved to reach similar quantities of tissue. This level of replication gives slack in the system for repeat extractions where sufficient quantities or qualities of data have not been achieved and also provides material for biobanking, enabling future expansion of results with new approaches (e.g., protein, metabolite analysis or new or improved genome/transcriptome sequencing technologies). We strongly recommend

getting in touch with the facility that is likely to complete the sequencing prior to specimen collection in the field to check their sample requirements (i.e. tube type, number of specimens required, if they accept samples preserved in ethanol or not, etc), as those vary depending on the facility and their experience in processing certain taxonomic groups.

Specimen Processing

As an organism is processed, it should be photographed alongside a tracking identifier (e.g., a SPECIMEN_ID) and alongside the barcodes of the tubes into which it is processed (Figure 3). These photographs are in addition to any that might be taken to document the living specimen and are useful for resolving sample tracking problems that can arise. We strongly encourage the use of barcoded tubes and scanning of these tubes rather than hand-writing identifiers on tubes and manual entry of identifiers into tracking systems as this is prone to error. For samples in the dozens or hundreds, this can be done with a simple single-tube scanner or even with a phone and an application like EpiCollect (https://five.epicollect.net). For larger projects processing many hundreds or thousands of specimens, rack scanners can be used to scan whole racks of barcoded tubes before sample processing.

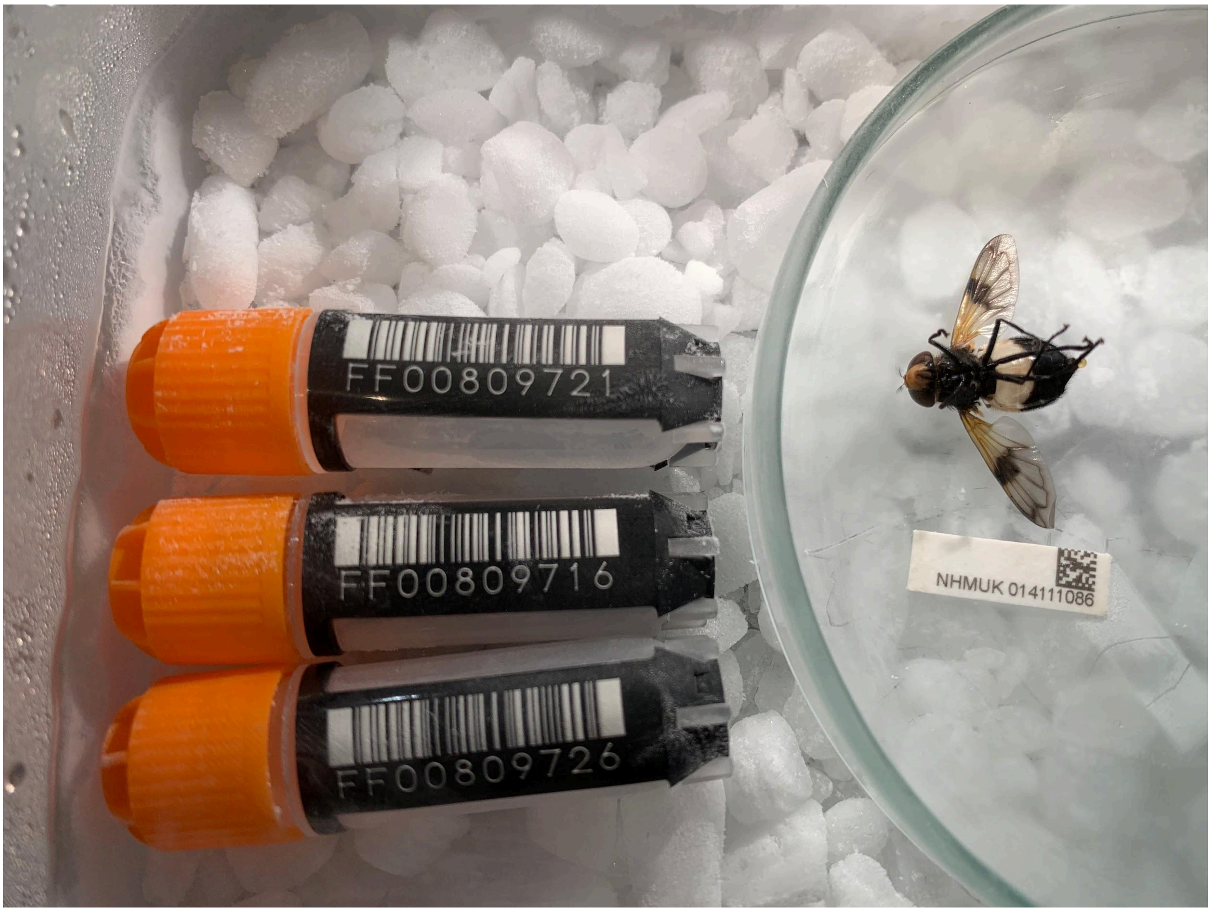

Figure 3. An example of the documentation that should occur as a sample is being processed. Here the SPECIMEN_ID (the NHM barcode under the fly) is photographed alongside the specimen and the barcoded tubes to which different samples of that specimen are destined. The metadata tracking sheet would thus have three entries for this fly, where collection-related information would be identical, but tissue type and tissue size would vary (e.g., head, thorax, and abdomen each in a separate tube). Photograph by MKNL.

Living specimens should be processed into tubes on dry ice and from that point forward, held at -80°C or below (e.g. in liquid nitrogen). Specimens that have died before processing tend to have damaged and degraded DNA and RNA, or can become highly contaminated with bacteria, and should not be used for reference genome generation. Specimens should be taken to a site where dry ice or liquid nitrogen are available, humanely euthanized, and rapidly processed into small lentil-sized pieces (Figure 4) while freezing, for example using a petri dish on dry ice and a scalpel.

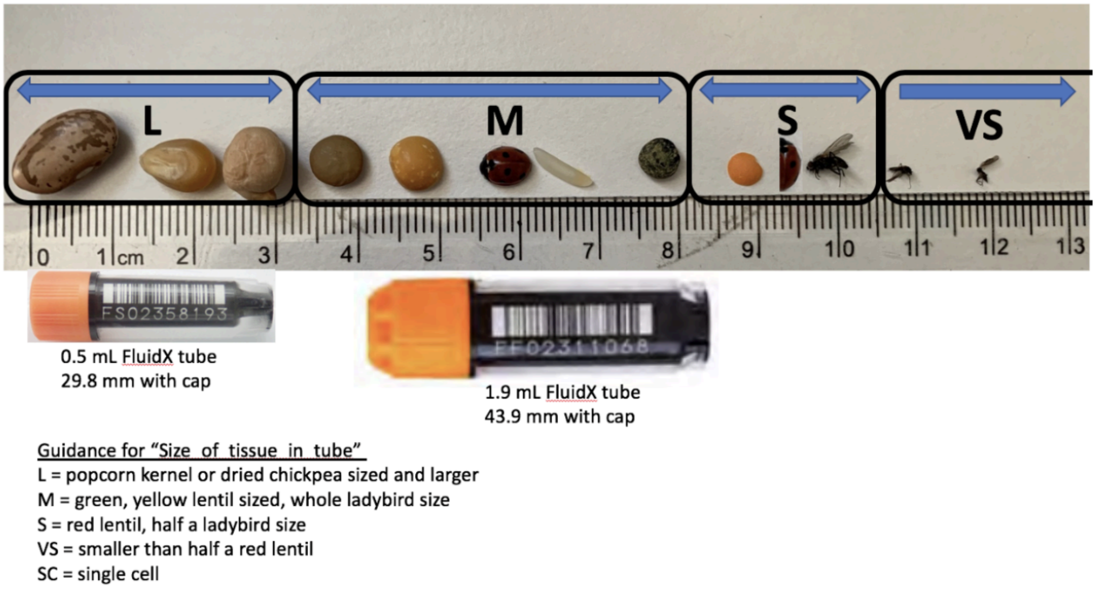

Figure 4. A scale showing the sizes of tissues that should typically be aimed for in a single tube. In some cases, (e.g. the flies in the Very Small (VS) category, there is no option to provide more tissue, so multiple separate specimens will be needed. The exact amount of tissue to supply depends on the taxon and the genome size, so it is best to discuss this in detail with the sequencing facility before beginning species collections. Facilities will not want to receive pieces of tissue much larger than the Large (L) size shown here as the tissue will be frozen solid and it is extremely difficult to break suitable sized pieces off without compromising the DNA or RNA integrity (e.g. by thawing). Plants will generally need substantially more tissue than is shown here. And as advised in the main text, for each species, we recommend aiming for 10 tubes, each containing a piece of tissue in the S-L range (typically 20-50 milligrams). Photograph by MKNL.

Small pieces of specimens sitting on dry ice have generally generated high-quality DNA as long as the freezing process was rapid. Small tissue pieces can then be placed into pre-chilled barcoded tubes, e.g. in Figure 3, the fly could be cut into head, thorax, and abdomen and each piece put in a separate tube. Currently, for animals and multicellular fungi, we recommend one piece of > 10 mg tissue per tube to support different workstreams without compromising the temperature of the remainder of the material through freeze-thaw cycles. Plants and multicellular algae should also be processed into small pieces to support rapid freezing, but larger volumes of tissue might be placed in tubes as up to ten times more tissue may be required to achieve adequate quantities of DNA for these groups. For many taxa where the majority of the specimen will be consumed in the process of data generation (e.g. most insects), it may be advisable to grind the whole organism to a fine powder in liquid nitrogen to avoid different data types being generated from different tissues (e.g., Hi-C data coming from the head, and long-read data coming from the abdomen, each tissue with distinct associated cobiont taxa).

Sequencing the very small

For organisms where achieving milligrams of tissue is not possible including meiofauna (i.e., animals < 1 mm in size) and unicellular eukaryotes, Ultra Low Input (ULI) protocols can be adopted to generate long-read data from single microscopic organisms. Protocols leveraging long-range PCR [11,12] or multiple displacement amplification [13,14] for microscopic organisms are available with caveats that DNA integrity and genome size are important considerations and these protocols may not perform well on large, highly repetitive genomes [12,14]. We advise that single specimens be used to generate long-read data, even for the smallest organisms. A second individual may be needed to produce a transcriptome (but see [12]). Commercially available cDNA library preparation kits enable obtaining high-quality transcriptome data from a single minute animal or even single cells (e.g., [15]). Coassembly of individual single amplified genomes (SAGs) has been moderately successful for some protist species, though not yet to the standards desired by EBP [16,17]. However, pooling several individuals (ideally related individuals of the same sex/mating type) may be necessary for Hi-C. The ULI approaches might not be sufficient for achieving EBP assembly quality standards, but at this point of technology development, it is the best that can be done for many microscopic taxa. When multiple individuals must be pooled for small organisms, species and collection sites should be chosen to ideally yield several specimens; these lots need to be collected at the same time and location, and carefully determined taxonomically to ascertain whether they belong to the same species. In the case of unicellular eukaryotes that are difficult to determine species, refraining from pooling cells will ensure that only a single species is sequenced. Ideally, images of the cell/organism sequenced should be taken for vouchering purposes.

Cold chain challenges

Situations in which preserving samples from living organisms without access to dry ice or liquid nitrogen are likely to increase in frequency as the EBP progresses. We are still learning which preservatives offer the best chance at successful long-read and long-range sequencing, and we recommend sharing successful protocols openly and early using the EBP protocols.io workspace (see below). As of now, we suggest if there is no possibility of rapid processing and preservation of a specimen from living to -80°C or below that samples are processed into small lentil-sized pieces in an excess (high preservative volume to tissue volume ratio) of 100% ethanol for HMW DNA and Hi-C, and RNAlater for RNA, and that

these are then stored at the coldest temperature possible. RNAlater-ICE does not produce a precipitate upon cooling and may be better than standard RNAlater for storage of RNA preserved material at below-freezing temperatures. Lower percentages of ethanol are not advised as they seem to result in more degraded DNA but this is still a grey area and different taxa may have different requirements. High-quality genomes have been generated from specimens stored in lower percentages of ethanol for long periods, but here we offer general guidance on best-known practices rather than what *might* work.

For small insects that may not require further processing and so be preserved whole, compromising the cuticle to permit ethanol penetration is critical for preserving HMW DNA [18] and has resulted in high-quality reference genomes even when insects were shipped for over one week at room temperature [19]. The ratio of tissue to preservative must also be considered as water in tissues may dilute the preservative. It is advised to change ethanol twice within 24 hours following preservation for tissues with high water content. We recommend a >20:1 preservative to tissue ratio. As soon as access to a -80°C freezer is available, the samples should be frozen and records should be kept on how long samples were held at room temperature. For dissected vertebrate tissue, if liquid nitrogen is not readily available, storage in a preservation liquid for up to one week at 4°C before flash-freezing in the lab has resulted in high quality HMW-DNA and Hi-C [20]. Experience has shown that preservation in media designed for nucleic acid protection (such as DNAGuard or RNALater) is not optimal for subsequent Hi-C sequencing, as they induce excessive trans interactions, likely because of disruption of nuclei [20]. Intensive further testing of preservatives and their ability to protect HMW DNA, RNA, and material suitable for Hi-C across the tree of life is likely to be an active area of development over the coming years, and sharing successful preservation protocols will be valuable for EBP ambitions (see below).

Often, specimens will need to be shipped to a different location for further work. Customs and import of biological material are often slow and there is a risk of losing precious material due to a loss in maintenance of the cold chain. To avoid this, proper legal documentation for export and import and associated metadata should accompany the specimens when shipped. Some couriers (e.g. World Courier and BioCair) offer dry ice top-up or dry shipper service for a fee. Tissues that do not remain frozen for their entire journey will not yield HMW DNA or high-quality RNA unless they are in a suitable preservative.

An EBP protocols.io community

Collection, extraction, and library generation protocols are as important to retain and share, according to the FAIR principles [21], as the sample-associated metadata. We recommend sharing collection, preservation, and extraction protocols in open-source repositories where a unique Digital Object Identifier (DOI) is assigned to every document. We advise including those DOIs in publications arising from the genomes produced under the EBP umbrella. As our knowledge about the biochemical and genetic makeup of previously understudied taxa is increasing, so will the knowledge base behind the appropriate handling of samples of these taxa and their genetic material. It will increase the reproducibility of research and provide an invaluable resource for the global community.

Biologists worldwide are already actively developing and releasing protocols for ethically collecting specimens, collecting comprehensive metadata, vouchering samples, preserving and processing samples, extracting RNA and HMW DNA, sequencing RNA and DNA, performing Hi-C, etc. These protocols are abundant but still primarily focused on a small number of taxonomic groups and are also typically hidden in research manuscripts rather than released as step-by-step protocols. As we learn what modifications or entirely different approaches work best for different taxonomic groups, we encourage open sharing of this information as early as possible. To assist with this, we have created an Earth BioGenome Project protocols.io workspace at https://www.protocols.io/workspaces/earth-biogenome-project. We recommend that the entire community publish their genome-relevant protocols at protocols.io (this is free and results in a citable DOI) and then link their protocols to the EBP workspace. To do this, users must join the EBP workspace and once their protocol is published, simply link it as shown in Figure 5.

In addition to contributing new protocols to the EBP workspace, we also recommend extensive commenting and forking of existing protocols. Commenting on existing protocols can help rapidly share information on successes and failures, as well as minor tips and recommendations that improve chances of success. Commenting and forking are easily achieved on any protocol through the "COMMENTS" and "COPY / FORK" clickable buttons that are present on every protocol. We suggest using comments to indicate if the protocol worked for specific species (include higher taxonomic information, e.g. "This protocol results

in high-quality HMW DNA for *Hapalochlaena lunulata*, an octopus. The DNA was successfully sequenced using PacBio HiFi". Forking can be used where an existing protocol has been modified more extensively to improve results for example for a particular tissue or taxon.

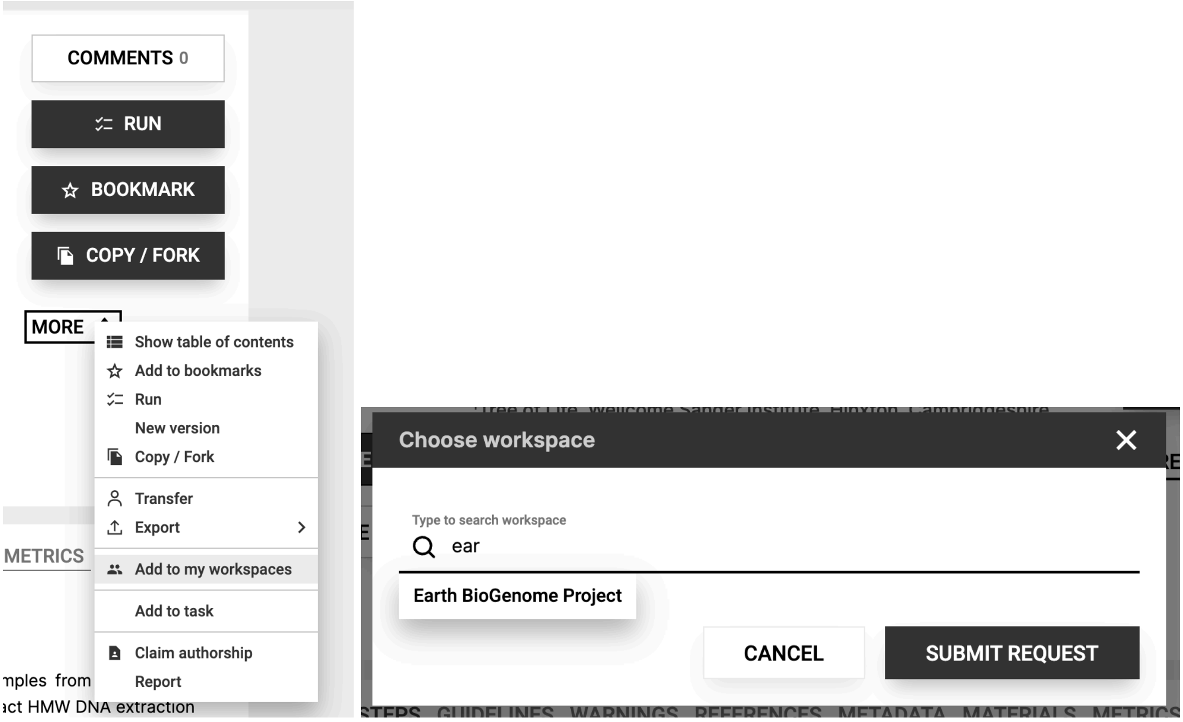

Figure 5. How to link protocols to the EBP workspace. Step 1. Join the Earth BioGenome Project Workspace at https://www.protocols.io/workspaces/earth-biogenome-project Step2. Click on the "MORE" menu as shown in the left panel above and select "Add to my workspaces". Step 3. Select "Earth BioGenome Project" as the workspace you would like to add the protocol to.

In the process of trying to go from specimen to high quality reference genome, we learn through unexpected successes and failures the tips and tricks that are not protocol-worthy, but still should be shared. If we had a way to share these anecdotes, we might save people treading down the same dead end roads. Or give them a tip that opens up huge possibilities. Therefore, we have created a community owned Google sheet called "EBP community collection of anecdotes" (https://tinyurl.com/EBP-Anecdotes). This is set up to simply collect experiences of anyone working the general area of reference genomes for biodiversity. We ask that the EBP community continue to populate this resource with their tips and tricks and we have provided some guidelines at the top of the document.

Fostering Inclusive Collaboration for Enhanced Impact

Strengthening collaboration and engagement across the biodiversity genomics stakeholder community will accelerate progress and maximize scientific impact and benefit sharing. The biodiversity genomics stakeholder community includes individuals from all relevant subdisciplines of biology: those traditionally engaged, such as geneticists, genomicists, and bioinformaticians; experts in taxonomy, natural history and ecological and evolutionary theory, including ecologists, evolutionary biologists, taxonomists, and systematists; and those often overlooked, such as individuals working in natural history collections who are involved in vouchering and specimen curation, staff at sequencing facilities who assist with project design and data generation, and those providing support in other aspects of project design, specimen acquisition, data generation, and curation.

The success of EBP is intrinsically linked to the collaborative efforts of scientists across a wide range of disciplines. Researchers working in natural history collections, at biobanks, taxonomists, systematists, genomicists, bioinformaticians, and support staff at sequencing facilities all play crucial roles. These professionals collect, identify, preserve, and maintain specimens, infer phylogenetic relationships, and study the evolution of traits and geographic distributions of organisms. Their work enables the genomic research community to access well-curated and accurately identified specimens, ensuring the correct identification and systematic placement of species-representative reference genomes. It is essential for all involved to acknowledge and strengthen these relationships, advocating for the vital work done by museum scientists, systematists, taxonomists, and technical support staff. Additionally, relevant taxonomic and systematic work, such as species descriptions and evolutionary context studies, and IDs of collection material examined, must be cited in genomics publications. Ensuring that individuals who collected and identified specimens are included as authors on resulting papers fosters a collaborative approach and gives credit where it is due [22].

Enhanced collaborations, e.g., among genomicists, systematists, taxonomists, museum scientists, bioinformaticians, and sequencing facility staff improve the scientific rigor of genomic research and recognize the critical contributions of experts in taxonomy, evolution and organismal biology [23,24]. Systematics and taxonomy provide an indispensable framework for genomics research. Experts in these fields often lead advancements in imaging, informatics, ecology, evolution, genetics, and genomics (e.g., [25–27]), and often

yield important new discoveries at the interface between traditional subdisciplines of biology (e.g., [28,29]). Presently, many groups of organisms are studied by only a few living taxonomic experts, making reliable identification challenging, at best. Alarmingly, limited taxonomic training in undergraduate curricula, fewer living expert taxonomists mentoring students and postdocs, and limited funding for taxonomic research stand to exacerbate this issue [24]. Collaborative efforts can provide valuable training for the next generation of integrative biodiversity scientists.

Close collaborations among genomicists, museum scientists and taxonomists can also prevent duplicative sampling efforts, minimize the ecological impact of specimen collection, conserve resources, and ensure efficient sampling. With genomic sequencing now more cost-effective, it is crucial to maximize the value of each collected specimen [30]. The genomics community, with its expertise in bioinformatics and data management, can significantly contribute to biodiversity informatics and digitization initiatives [31]. Researchers collaborating with museums can advocate for best practices in specimen preservation that will facilitate future genomic work and ensure the deposition of voucher specimens in public collections [32]. Of course, many scientists in the field of biodiversity genomics also engage in systematics and taxonomy and/or work in museum settings, demonstrating the interdisciplinary nature of modern genomics research (e.g., [33]).

All members of the EBP stakeholder community, including genomicists, systematists, taxonomists, museum scientists, bioinformaticians, sequencing facility staff, and others, play important interdependent roles in this endeavor. Strengthening collaboration among these communities will enhance the quality and impact of research, ensuring it is ethical, sustainable, and inclusive. Through such partnerships we can more fully and effectively explore and preserve the diversity of life on Earth for generations to come.

Looking to the Future

Current best practice assembly guidelines are to generate a combination of data types including long-read (PacBio HiFi and/or ultra-long ONT), long-range (Hi-C), and RNAseq (Illumina short read, PacBio Kinnex, or ONT cDNA-PCR) data from the same specimen wherever possible, aiming for the heterogametic sex when this is relevant. Typically, separate samples are used for these different applications, but we should be developing protocols that support minimal extraction of material sufficient for any of these types of

data generation (e.g., nuclei co-extracted with RNA). Furthermore, in all current extraction efforts, we discard material that we might one day look back on and regret, such as proteins and metabolites. While data generation from these materials is currently out of scope, this is unlikely to be true in years to come. Retaining relevant material to add data layers to the high-quality reference genomes would be prudent. Thus, for specimens where samples are available in excess, considerations should be given to preserving replicate samples and appropriate storage to future-proof these samples as much as possible. For specimens where all material is used in data generation, perhaps typically discarded supernatants should be retained for future investigations.

As sequencing proceeds into phases comprising more fine-scale taxonomic coverage, we foresee that getting specimens identified *in vivo* before euthanizing and freezing will be challenging for many species-rich but poorly known taxa, including most invertebrate groups. Reliable identifications often depend on careful examination under the microscope by experts, of which there are typically only a few for entire taxonomic families; this would require holding the specimens out of the cold chain for a length of time that could compromise the DNA and RNA. This highlights the importance of appropriately preserved morphological voucher specimens and imaging. Further, advances in DNA barcoding reference libraries can be of enormous importance, so that specimens can be collected first and identified later. One possibility would be to have a fourth tissue sampling used initially only for DNA barcoding to attempt identification by comparing the sequence to available public databases. Such identifications would then inform the decisions about whether a sample should be further processed for genomic sequencing.

We also encourage activities that simplify and streamline standard operating procedures and protocols to make it easier to “containerize” extraction, sequencing, and assembling activities. Containerization means a world in which a simple portable lab could house everything needed to go from sample to sequence and would build capacity in the Global South, in the nations that often harbor the greatest biodiversity. This relieves pressure on Nagoya Permit requirements and the budget spent on expensive shipping costs to maintain the cold-chain, but more importantly, it is better for global science.

Numerous unexplored opportunities also lie within the realm of AI and automation, waiting to be unlocked and harnessed. AI-driven robotic systems could increase efficiency by automating sample collection and optimizing workflows, significantly reducing the time and

resources required (e.g. [34]). This integration could enhance precision and accuracy through AI-based quality control and precise data annotation, ensuring only high-quality samples contribute to genome sequencing. Costs could thus be reduced through labor savings and resource optimization, while the speed of genome sequencing could be accelerated by automated processing and parallelization of samples. Furthermore, AI could be used to facilitate intelligent sampling strategies, promoting diversity in collected samples, and enabling real-time monitoring of environmental conditions. Integrating biodiversity data into centralized databases and advanced analytics enhances data accessibility and analysis. Overall, the synergy of AI and automation may improve the quality and speed of reference genome generation and contribute to a deeper understanding of biodiversity for applications in conservation, ecology, and evolutionary biology.

Abbreviations

EBP = Earth BioGenome Project

GoaT = Genomes on a Tree

Gb = Gigabase

INSDC = International Nucleotide Sequence Database Collection

ABS = Access and Benefit Sharing

BOLD = Barcode of Life Database

ToLID = Tree of Life Identification

Taxid = Taxonomic identification

MTA = Material Transfer Agreement

HMW = High Molecular Weight

PacBio = Pacific Biosciences

ONT = Oxford Nanopore Technologies

Declarations

Data Availability: No data was generated or used in the drafting of this article.

Competing Interests: The authors declare that they have no competing interests.

Funding: Authors MKNL, MB, and CGSC are funded by the Wellcome Trust quinquennial award to the Wellcome Sanger Institute (grant number 220540/Z/20/A). OVP is funded by the SciLifeLab (Science for Life Laboratory, Sweden), and the Swedish Research Council (RFI/VR). KMK is funded by National Science Foundation grants 1846174, 2138994, 2321308, and 2001303. VR is funded by the NIH Common Fund Award / NHGRI Grant Number U24HG006941 and National Institutes of Health (OD), National Institution of Biomedical Imaging and Bioengineering, and NIH award number 1 U2C EB 032224 - 01. DDM is funded by National Science Foundation grants 2110053 and 1937815. JGW is funded by the National Science Foundation grant DBI-2119963, BII: Mechanisms of Cellular Evolution. AKC is supported by the U.S. Department of Agriculture, Agricultural Research Service (USDA-ARS), Bee Research Laboratory in-house appropriated research project 8042-21000-291-000-D.

Authors' Contributions: M.K.N.L. drafted the original manuscript. The EBP Sample Collection and Processing sub-Committee members conceptualized the manuscript. All authors contributed to review and editing. EBP Sample Collection and Processing sub-Committee as of 2024: Chair Mara K. N. Lawniczak and all members (listed alphabetically): Jonas Astrin, Anna Childers, Kevin Kocot, Duane McKenna, Olga Pettersson, Verena Ras, Bernardo Santos, and Jeremy Wideman.

Acknowledgements: The authors thank Harris Lewin and Federica di Palma for comments.

References

1. Blaxter M, Archibald JM, Childers AK, Coddington JA, Crandall KA, Di Palma F, et al. Why sequence all eukaryotes? Proc Natl Acad Sci U S A. 2022;119. doi:10.1073/pnas.2115636118

2. Challis R, Kumar S, Sotero-Caio C, Brown M, Blaxter M. Genomes on a Tree (GoaT): A versatile, scalable search engine for genomic and sequencing project metadata across the eukaryotic tree of life. Wellcome Open Res. 2023;8: 24. doi:10.12688/wellcomeopenres.18658.1

3. Mc Cartney AM, Head MA, Tsosie KS, Sterner B, Glass JR, Paez S, et al. Indigenous peoples and local communities as partners in the sequencing of global eukaryotic biodiversity. NPJ Biodivers. 2023;2: 8. doi:10.1038/s44185-023-00013-7

4. Böhne A, Fernández R, Leonard JA, McCartney AM, McTaggart S, Melo-Ferreira J, et al. Contextualising samples: supporting reference genomes of European biodiversity through sample and associated metadata collection. NPJ Biodivers. 2024;3: 26. doi:10.1038/s44185-024-00053-7

5. Sherkow JS, Barker KB, Braverman I, Cook-Deegan R, Durbin R, Easter CL, et al. Ethical, legal, and social issues in the Earth BioGenome Project. Proc Natl Acad Sci U S A. 2022;119. doi:10.1073/pnas.2115859119

6. Lawniczak MKN, Davey RP, Rajan J, Pereira-da-Conceicoa LL, Kilias E, Hollingsworth PM, et al. Specimen and sample metadata standards for biodiversity genomics: a proposal from the Darwin Tree of Life project. Wellcome Open Res. 2022;7: 187. doi:10.12688/wellcomeopenres.17605.1

7. Blaxter M, Pauperio J, Schoch C, Howe K. Taxonomy Identifiers (TaxId) for Biodiversity Genomics: a guide to getting TaxId for submission of data to public databases. 2024. doi:10.12688/wellcomeopenres.22949.1

8. Corrales C, Astrin JJ. Biodiversity Biobanking – a Handbook on Protocols and Practices. AB. 2023;1: Advanced Books. doi:10.3897/ab.e101876

9. ISBER.. Available: https://www.isber.org/page/BPR

10. Frankham R. Genetic adaptation to captivity in species conservation programs. Mol Ecol. 2008;17: 325–333. doi:10.1111/j.1365-294X.2007.03399.x

11. Schneider C, Woehle C, Greve C, D'Haese CA, Wolf M, Hiller M, et al. Two high-quality de novo genomes from single ethanol-preserved specimens of tiny metazoans (Collembola). Gigascience. 2021;10. doi:10.1093/gigascience/giab035

12. Laumer C. Picogram input multimodal sequencing (PiMmS). 2023. Available: https://www.protocols.io/view/picogram-input-multimodal-sequencing-pimms-rm7vzywy5lx1/v1

13. Lee Y-C, Ke H-M, Liu Y-C, Lee H-H, Wang M-C, Tseng Y-C, et al. Single-worm long-read sequencing reveals genome diversity in free-living nematodes. Nucleic Acids Res. 2023;51: 8035–8047. doi:10.1093/nar/gkad647

14. Roberts NG, Gilmore MJ, Struck TH, Kocot KM. Multiple Displacement Amplification Facilitates SMRT Sequencing of Microscopic Animals and the Genome of the Gastrotrich Lepidodermella squamata (Dujardin, 1841). bioRxiv. 2024. p. 2024.01.17.576123. doi:10.1101/2024.01.17.576123

15. Varney RM, Funch P, Kocot KM, Sørensen MV. A new species of Echinoderes (Cyclorhagida: Echinoderidae) from the San Juan Islands, Washington State, USA, and insights into the kinorhynch transcriptome. Zool Anz. 2019;282: 52–63. doi:10.1016/j.jcz.2019.06.003

16. Mangot J-F, Logares R, Sánchez P, Latorre F, Seeleuthner Y, Mondy S, et al. Accessing the genomic information of unculturable oceanic picoeukaryotes by combining multiple single cells. Sci Rep. 2017;7: 41498. doi:10.1038/srep41498

17. Sørensen MES, Zlatogursky VV, Onuţ-Brännström I, Walraven A, Foster RA, Burki F. A novel kleptoplastidic symbiosis revealed in the marine centrohelid Meringosphaera with evidence of genetic integration. Curr Biol. 2023;33: 3571–3584.e6. doi:10.1016/j.cub.2023.07.017

18. Teltscher F, Lawniczak M. Squishing insects for preservation of HMW DNA in the field. 2023. Available: https://www.protocols.io/view/squishing-insects-for-preservation-of-hmw-dna-in-t-4r3l2224jl1y/v1

19. Nsango SN, Ayala D, Agbor J-P, Johnson HF, Heaton H, Wagah MG, et al. A chromosomal reference genome sequence for the malaria mosquito, *Anopheles nili*, Theobald, 1904. 2024. doi:10.12688/wellcomeopenres.23198.1

20. Dahn HA, Mountcastle J, Balacco J, Winkler S, Bista I, Schmitt AD, et al. Benchmarking ultra-high molecular weight DNA preservation methods for long-read and long-range sequencing. Gigascience. 2022;11: giac068. doi:10.1093/gigascience/giac068

21. Wilkinson MD, Dumontier M, Aalbersberg IJJ, Appleton G, Axton M, Baak A, et al. The FAIR Guiding Principles for scientific data management and stewardship. Sci Data. 2016;3: 160018. doi:10.1038/sdata.2016.18

22. Meier R. Citation of taxonomic publications: the why, when, what and what not: Species citations. Syst Entomol. 2016;42. doi:10.1111/syen.12215

23. Drew LW. Are We Losing the Science of Taxonomy? As need grows, numbers and training are failing to keep up. Bioscience. 2011;61: 942–946. doi:10.1525/bio.2011.61.12.4

24. Löbl I, Klausnitzer B, Hartmann M, Krell F-T. The Silent Extinction of Species and Taxonomists—An Appeal to Science Policymakers and Legislators. Diversity . 2023;15: 1053. doi:10.3390/d15101053

25. Faulwetter S, Vasileiadou A, Kouratoras M, Thanos Dailianis, Arvanitidis C. Micro-computed tomography: Introducing new dimensions to taxonomy. Zookeys. 2013; 1–45. doi:10.3897/zookeys.263.4261

26. Meyer C, Duffy E, Collins A, Paulay G, Wetzer R. The U.S. Ocean Biocode. Mar Technol Soc J. 2021;55: 140–141. doi:10.4031/MTSJ.55.3.33

27. Pante E, Schoelinck C, Puillandre N. From integrative taxonomy to species description: one step beyond. Syst Biol. 2015;64: 152–160. doi:10.1093/sysbio/syu083

28. McKenna DD, Shin S, Ahrens D, Balke M, Beza-Beza C, Clarke DJ, et al. The evolution and genomic basis of beetle diversity. Proc Natl Acad Sci U S A. 2019;116: 24729–24737. doi:10.1073/pnas.1909655116

29. Stiller J, Feng S, Chowdhury A-A, Rivas-González I, Duchêne DA, Fang Q, et al. Complexity of avian evolution revealed by family-level genomes. Nature. 2024;629:

851–860. doi:10.1038/s41586-024-07323-1

30. Funk VA. Collections-based science in the 21st Century. J Syst Evol. 2018;56: 175–193. doi:10.1111/jse.12315

31. Guralnick R, Hill A. Biodiversity informatics: automated approaches for documenting global biodiversity patterns and processes. Bioinformatics. 2009;25: 421–428. doi:10.1093/bioinformatics/btn659

32. Colella JP, Stephens RB, Campbell ML, Kohli BA, Parsons DJ, Mclean BS. The Open-Specimen Movement. Bioscience. 2021;71: 405–414. doi:10.1093/biosci/biaa146

33. Droege G, Barker K, Astrin JJ, Bartels P, Butler C, Cantrill D, et al. The Global Genome Biodiversity Network (GGBN) Data Portal. Nucleic Acids Res. 2014;42: D607–12. doi:10.1093/nar/gkt928

34. Wührl L, Pylatiuk C, Giersch M, Lapp F, von Rintelen T, Balke M, et al. DiversityScanner: Robotic handling of small invertebrates with machine learning methods. Mol Ecol Resour. 2022;22: 1626–1638. doi:10.1111/1755-0998.13567

Manuscript

Click here to access/download;Personal Cover;GigaScienceSubmission.pdf

Mara K. N. Lawniczak – Senior Group Leader
Tree of Life
Wellcome Sanger Institute
Hinxton CB10 1SA
United Kingdom
phone: +44 1223 494 821
email: mara@sanger.ac.uk

18 October 2024

Dear Gigascience Editors

On behalf of the Earth BioGenome Project, we are submitting our manuscript offering current best practice guidance for sample collection and processing. Throughout, this manuscript also considers challenges and future needs. I had a brief pre-submission enquiry with Scott Edmunds to confirm that this could be an appropriate manuscript for your journal, and we would love to see it published there.

I am aware that the links throughout are not referenced properly. I will be happy to fix this upon revision, but for review, we find that having the embedded link right there as you read is much easier than needing to go back and forth to references. It would be great if this is accepted to consider if that might be possible in the published version (alongside adding the links to references) given how many embedded links are in the manuscript.

Thank you for consideration.

Best

Mara Lawniczak
