# Supplementary material for: Best-practice guidance for Earth BioGenome Project sample collection and processing: progress and challenges in biodiverse reference genome creation
Source: Gigascience. 2025 May 29;14:giaf041. doi: 10.1093/gigascience/giaf041 (PMC12121479; doi:10.1093/gigascience/giaf041)
Supplement: giaf041_GIGA-D-24-00457_Revision_1 [file giaf041_giga-d-24-00457_revision_1.pdf]

GigaScience

Best Practice Guidance for Earth BioGenome Project Sample Collection and Processing: Progress and Challenges in Biodiverse Reference Genome Creation

--Manuscript Draft--

**Manuscript Number:**	GIGA-D-24-00457R1
**Full Title:**	Best Practice Guidance for Earth BioGenome Project Sample Collection and Processing: Progress and Challenges in Biodiverse Reference Genome Creation
**Article Type:**	Review
**Funding Information:**	Wellcome Trust (220540/Z/20/A) — Dr Mark Blaxter
**Abstract:**	The Earth BioGenome Project (EBP) has the extremely ambitious goal of generating, at scale, high quality reference genomes across the entire Tree of Life [1]. Currently in its first phase, the project is targeting family-level representatives and is progressing rapidly. Here we outline recommended standards and considerations in sample acquisition and processing for those involved in biodiverse reference genome creation. As a living document, these standards and recommendations will evolve with advances in related processes. Additionally, we discuss the challenges raised by the ambitions for later phases of the project, highlighting topics related to sample collection and processing that require further development.
**Corresponding Author:**	Mara Lawniczak<br>Wellcome Sanger Institute<br>Hinxton, UNITED KINGDOM
**Corresponding Author Secondary Information:**	
**Corresponding Author's Institution:**	Wellcome Sanger Institute
**Corresponding Author's Secondary Institution:**	
**First Author:**	Mara Lawniczak
**First Author Secondary Information:**	
**Order of Authors:**	Mara Lawniczak
	Kevin Kocot
	Jonas Astrin
	Mark Blaxter
	Cibele Sotero-Caio
	Katharine Barker
	Anna Childers
	Jonathan Coddington
	Paul Davis
	Kerstin Howe
	Warren Johnson
	Duane McKenna
	Jeremy Wideman
	Bernardo Santos
**Order of Authors Secondary Information:**	
**Response to Reviewers:**	This is in previous as "cover letter"

Powered by Editorial Manager® and ProduXion Manager® from Aries Systems Corporation

Additional Information:	
Question	Response
Are you submitting this manuscript to a special series or article collection?	No
**Experimental design and statistics**<br><br>Full details of the experimental design and statistical methods used should be given in the Methods section, as detailed in our Minimum Standards Reporting Checklist. Information essential to interpreting the data presented should be made available in the figure legends.<br><br>Have you included all the information requested in your manuscript?	No
If not, please give reasons for any omissions below.<br><br>as follow-up to "**Experimental design and statistics**<br><br>Full details of the experimental design and statistical methods used should be given in the Methods section, as detailed in our Minimum Standards Reporting Checklist. Information essential to interpreting the data presented should be made available in the figure legends.<br><br>Have you included all the information requested in your manuscript?<br><br>"	N/A
**Resources**<br><br>A description of all resources used, including antibodies, cell lines, animals and software tools, with enough information to allow them to be uniquely identified, should be included in the Methods section. Authors are strongly encouraged to cite Research Resource	No

Identifiers (RRIDs) for antibodies, model organisms and tools, where possible.<br><br>Have you included the information requested as detailed in our Minimum Standards Reporting Checklist?	
If not, please give reasons for any omissions below.<br><br>as follow-up to "**Resources**<br><br>A description of all resources used, including antibodies, cell lines, animals and software tools, with enough information to allow them to be uniquely identified, should be included in the Methods section. Authors are strongly encouraged to cite Research Resource Identifiers (RRIDs) for antibodies, model organisms and tools, where possible.<br><br>Have you included the information requested as detailed in our Minimum Standards Reporting Checklist?<br><br>"	N/A
**Availability of data and materials**<br><br>All datasets and code on which the conclusions of the paper rely must be either included in your submission or deposited in publicly available repositories (where available and ethically appropriate), referencing such data using a unique identifier in the references and in the "Availability of Data and Materials" section of your manuscript.<br><br>Have you have met the above requirement as detailed in our Minimum Standards Reporting Checklist?	No
If not, please give reasons for any omissions below.	N/A

as follow-up to "**Availability of data and materials**<br><br>All datasets and code on which the conclusions of the paper rely must be either included in your submission or deposited in publicly available repositories (where available and ethically appropriate), referencing such data using a unique identifier in the references and in the "Availability of Data and Materials" section of your manuscript.<br><br>Have you have met the above requirement as detailed in our Minimum Standards Reporting Checklist?<br><br>"	
GigaScience has policies and guidelines in place for the use of generative AI-writing tools such as ChatGPT. If you have used such writing tools to assist with writing the manuscript this must be declared and cited in the text. Authors should not list AI-writing tools and other AI-assisted technologies as an author or co-author and should acknowledge that they are fully responsible for text generated or refined by AI-writing tools.<p><br><br>A summary of use (particularly in the introduction or among methods) needs to be included at the end of the paper, and the outputs should also be included as a supplementary file hosted in GigaDB or other open repositories. Please read our guidelines for more information. <p><br><br>By submitting to GigaScience, you are aware of the journal's AI-writing tools policy, and if you have declared use of such tools below, you have acknowledged this where appropriate in your manuscript and have made a summary of use and outputs available. <p><br><b>AI-assisted writing tools have been used in the preparation of this manuscript?	No

Click here to access/download;Manuscript;Best Practices.docx

Best Practice Guidance for Earth BioGenome Project Sample Collection and Processing: Progress and Challenges in Biodiverse Reference Genome Creation

Mara K. N. Lawniczak[1], Kevin M. Kocot[2], Jonas J. Astrin[3], Mark Blaxter[1], Cibele G. Sotero-Caio[1], Katharine B. Barker[4], Anna K. Childers[5], Jonathan Coddington[4], Paul Davis[1], Kerstin Howe[1], Warren E. Johnson[4], Duane D. McKenna[6,7], Jeremy Wideman[8], Olga Vinnere Pettersson[9], Verena Ras[10,11], Bernardo F. Santos[12], and the Earth BioGenome Project Samples and Processing sub-Committee[13]

1. Tree of Life, Wellcome Sanger Institute, Hinxton, CB10 1SA United Kingdom
2. Department of Biological Sciences & Alabama Museum of Natural History, University of Alabama, Tuscaloosa, AL, USA
3. Leibniz Institute for the Analysis of Biodiversity Change, Museum Koenig, Bonn, Germany
4. National Museum of Natural History, Smithsonian Institution, Washington, DC, USA
5. USDA, Agricultural Research Service, Beltsville Agricultural Research Center, Bee Research Laboratory, 10300 Baltimore Avenue, Beltsville, MD 20705, USA
6. Department of Biological Sciences, University of Memphis, Memphis, TN 38152, USA
7. Center for Biodiversity Research, University of Memphis, Memphis, TN 38152, USA
8. Biodesign Center for Mechanisms of Evolution, School of Life Sciences, Arizona State University, Tempe, AZ, 85287, USA
9. SciLifeLab, National Genomics Infrastructure, Department of Immunology, Genetics and Pathology, Uppsala University, Uppsala, SE-751 05, Sweden
10. Computational Biology Division, Department of Integrative Biomedical Sciences, University of Cape Town, Cape Town, South Africa.
11. Department of Biodiversity and Conservation Biology, University of the Western Cape, Cape Town, South Africa.
12. Museum für Naturkunde, Leibniz Institute for Evolution and Biodiversity Science, Center for Integrative Biodiversity Discovery, Invalidenstraße 43, Berlin 10115, Germany

13. https://www.earthbiogenome.org/sample-collection-processing

ABSTRACT

The Earth BioGenome Project (EBP) has the extremely ambitious goal of generating, at scale, high quality reference genomes across the entire Tree of Life [1]. Currently in its first phase, the project is targeting family-level representatives and is progressing rapidly. Here we outline recommended standards and considerations in sample acquisition and processing for those involved in biodiverse reference genome creation. These standards and recommendations will evolve with advances in related processes. Additionally, we discuss the challenges raised by the ambitions for later phases of the project, highlighting topics related to sample collection and processing that require further development.

BACKGROUND

The Earth BioGenome Project (EBP) comprises a network of local, continental, and taxon focused projects (https://www.earthbiogenome.org/affiliated-project-networks), all of which are contributing to the vision that reference genomes across the Tree of Life have and will have huge impact on our understanding of the world around us and on planetary health. The EBP is currently in its first phase, with many projects already contributing and many more projects that may be initiated in the near future. EBP Phase I proposes the sequencing of a representative species for all of the ~9,500 described eukaryotic families (see https://www.catalogueoflife.org/). The EBP is a collective effort and as such, there is no "EBP Phase I target species list". Instead the EBP member projects are encouraged to create their target species lists to reach this goal collaboratively. For the majority of Phyla, a genome is available but many of these do not reach chromosomal level (Figure 1). With

respect to the Phase I goal, as of October 2024, there are genomes for 3000 species from over 1000 families, of which over 2000 species from over 750 families have chromosome level assemblies. Thus, significant work remains to complete Phase I. This document aims to provide recommendations for any project that is generating reference genomes.

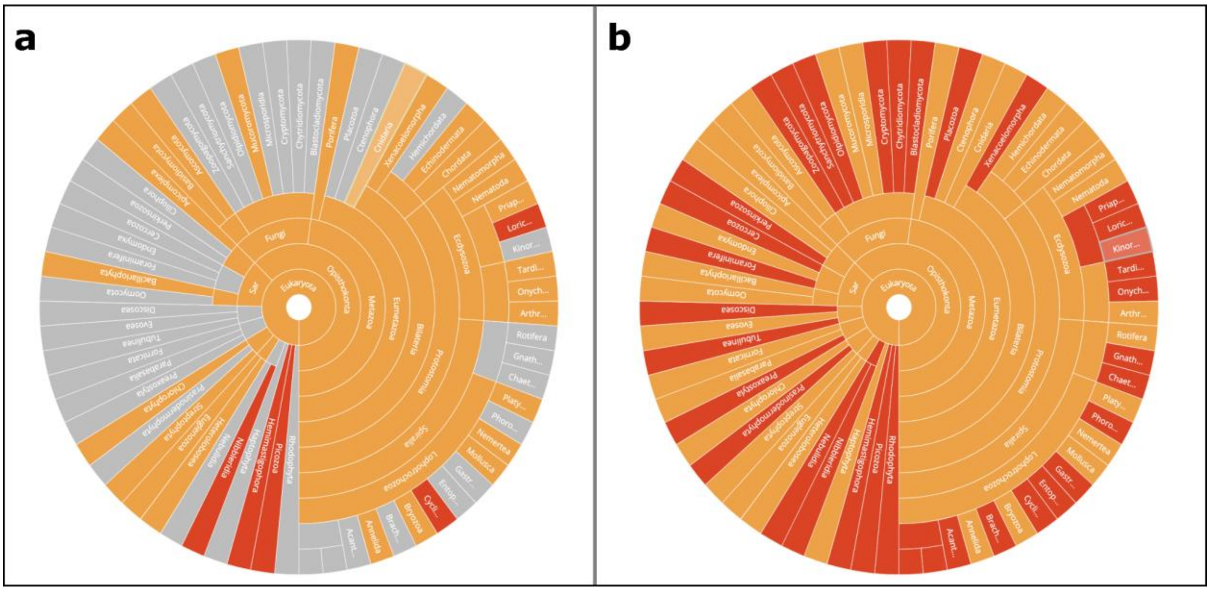

Figure 1. Genome sequencing across Eukaryotic phyla from NCBI taxonomy. a. All assembly levels: red - no available genomes; orange - at least 1 descendent sequenced by the EBP; grey - at least 1 descendent has an available genome. GoaT link for interactive tree. b. In orange are phyla with at least 1 EBP-quality assembly and in red, those with no available EBP-quality genomes. GoaT link for interactive tree.

Recommendations for success in sampling for biodiversity genomics

Here, we list general factors to consider as projects set out to select their target species, and then we discuss at more length some additional considerations. While this list is focused on EBP Phase 1, most of the recommendations are advisable even for projects that already have a clear species target list. This list is focused on larger multicellular organisms or smaller species that are easily cultured, but we discuss below ultra-low input approaches for protists and other species where physical size is a limitation. General factors to consider when suggesting a species representative towards the EBP Phase I goal include:

- **Community value**: Species should be of broad community use and value including species that are of economic or ecological value, are of conservation concern, have specific scientific interest, or have iconic status. Community value could be assessed through surveys oriented towards target communities (e.g.: https://tinyurl.com/dtol-suggest for the Darwin Tree of Life project).
- **Permissions and availability**: Sampling should be achievable, considering permissions and ethical and legal collection requirements, as well as ease of collection (geography, collecting method).
- **Publicly registered:** The species should be registered with its current name and taxonomy in a publicly available database (we recommend the NCBI Taxonomy Database https://www.ncbi.nlm.nih.gov/taxonomy) and assigned a numeric identifier to assist with tracking taxonomic changes over time. This is covered in more detail below.
- **Taxonomic stability & representation**: The taxon should not be subject to current disagreement and revision. From a taxonomic perspective, it is preferable to sample the type genus of the family, or at least a taxon from the type genus subfamily. The species should be generally considered a “good” biological species (not from a known species complex) and if possible, sampled from or near the type locality.
- **Voucher availability:** If the organism is large, multiple subsamples should be taken for tissue and nucleic acid biobanking. For smaller organisms that are likely to be

consumed in genome generation, ideally several additional individuals from the same time and place would be collected for morphological and molecular vouchering (e.g. biobanking of DNA, RNA, and/or tissue). Likewise, photographic documentation of living specimens should be undertaken whenever possible.

Additional factors to consider for multicellular organisms include:

- **Genome size, ploidy, sex, and life stage(s)**: Where genome sizes and/or ploidy are known (estimates are available via Genomes on a Tree (GoaT); see below), prioritize species with smaller, diploid genomes. This is because data generation costs will fall and our ability to assemble high repeat content genomes will improve in future EBP phases. Where sex chromosomes are understood, selecting the heterogametic sex if possible provides a more complete view of the genome. For species with haplodiploid sex determination, the haploid sex should be chosen.
- **Physical sample size**: We recommend a minimal requirement of ten samples, each weighing more than 10 milligrams per 1 Gb of genome size, for animals and multicellular / culturable fungi, culturable protists and 100 milligrams per 1 Gb of genome size for plants and multicellular algae. A minimum of three samples is suggested to support the three platforms of long read, chromatin conformation (Hi-C), and transcriptome (RNAseq) sequencing. Depending on genome size, amplification-based long-read sequencing approaches may still result in a high-quality reference genome for organisms where this is not possible.

The considerations listed above are not intended to be a “must have” list and each project will need to determine what is important for their particular aims. For example, one project might deem completing the genome of a single 9 Gb locust species to be too expensive when those funds could support genomes for 20 species, but another project might deem the locust genome extremely important. In other words, the prioritization of the considerations listed above should be made by each project independently.

In addition to the considerations listed above, there are a number of other areas key to successfully and ethically contributing to EBP goals. Here we set out considerations and

recommendations in each of these areas. The guidance focuses on what should be completed for “straightforward” species/specimens – those that are identifiable to species and macroscopic. This is because we are still in the early phase of the project, and when selecting species representatives from families, projects can often prioritise “straightforward” species. However, guidance for the more challenging microscopic or even single celled organisms is also covered later in the manuscript.

EBP Phase I family-representative target species: openly collating community proposals and genomes underway

As the technology needed to generate high quality reference genomes is improving and becoming increasingly globally accessible, global coordination to avoid duplication of effort (i.e., sequencing the same species) where possible is important. Multiple species for each family should be proposed and collected to provide greater flexibility in achieving Phase I goals. As much as possible, this process should be globally visible, with species underway and their associated projects made clear. All projects should use the Genomes on a Tree (GoaT) system (https://goat.genomehubs.org/, [2]) to share their target lists and progress. GoaT has a searchable interface that lists a constantly updated database of progress reported from ongoing reference genome projects and also provides direct and inferred values for genome size, ploidy, and chromosome number. These are extremely useful metrics when selecting target species.

In addition to assisting with global coordination, contributing lists of target species to GoaT allows genomes sequenced through these projects to be tracked and counted both to local and EBP Phase I goals. These target lists declare intent, and as such, are likely to undergo revision as projects proceed due to, for example, challenges with acquiring specific target species or extracting adequate DNA and RNA from them. We recommend that larger projects create and maintain a live file listing their target species, identifying taxa selected as EBP family representatives (Figure 2a), and supply the file URL to the GoaT data curators. The format for the file is given in https://goat.genomehubs.org/submissions, and a

2024 version is presented at http://tinyurl.com/goat-submission-template. GoaT archives the submitted file and processes it for display. Larger funded projects are often open to receiving suggestions for target taxa and we encourage genome users to request particular species from the most relevant existing EBP projects (e.g. for an African species, contact AfricaBP).

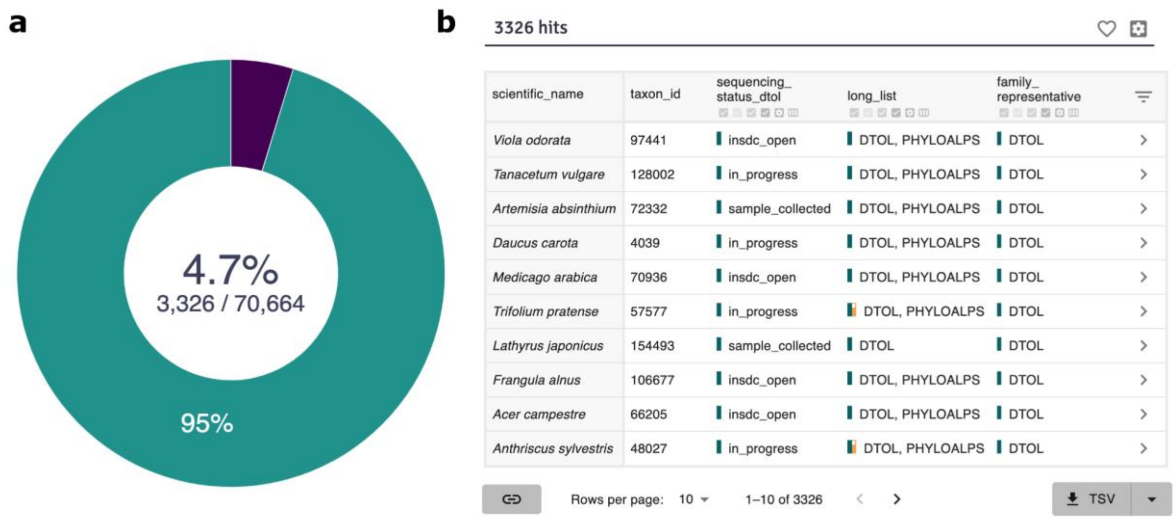

scientific_name	taxon_id	sequencing_status_dtol	long_list	family_representative
Viola odorata	97441	insdc_open	DTOL, PHYLOALPS	DTOL
Tanacetum vulgare	128002	in_progress	DTOL, PHYLOALPS	DTOL
Artemisia absinthium	72332	sample_collected	DTOL, PHYLOALPS	DTOL
Daucus carota	4039	in_progress	DTOL, PHYLOALPS	DTOL
Medicago arabica	70936	insdc_open	DTOL, PHYLOALPS	DTOL
Trifolium pratense	57577	in_progress	DTOL, PHYLOALPS	DTOL
Lathyrus japonicus	154493	sample_collected	DTOL	DTOL
Frangula alnus	106677	insdc_open	DTOL, PHYLOALPS	DTOL
Acer campestre	66205	insdc_open	DTOL, PHYLOALPS	DTOL
Anthriscus sylvestris	48027	in_progress	DTOL, PHYLOALPS	DTOL

Figure 2. Example of a search for family representatives on GoaT. a. Report of Darwin Tree of Life (DToL) species proposed as family representatives out of all > 60K species on the DToL wishlist. b. List of species from the report in (a.) with additional columns displaying target (scope) overlaps when present (long_list = DToL, PHYLOALPS) and current sequencing status within the DToL genome production pipeline. GoaT search link.

GoaT can import lists of intent and progress for smaller projects if the project is registered under an umbrella bioproject ID on INSDC and ideally linked to at least one major EBP initiative. Projects should contact one of the major initiatives that best fit their scope to be listed as a contributing lab. The main contact for each project can be found in each project-dedicated page at https://goat.genomehubs.org/projects. Target lists and contact information on GoaT are public and resolution of any overlaps (e.g., Figure 2b) can be initiated by either project via the contact information provided.

Use cases for exploring available EBP data and also identifying sequencing gaps in taxonomic groups can be found at https://goat.genomehubs.org/help/use_cases. Summary information and live progress reports are available as a project-dedicated page for all EBP affiliates at https://goat.genomehubs.org/projects. Each project page contains duplication checkers, where the overlap of target species or progress can be cross-referenced among EBP affiliates. Projects should take advantage of the duplication checkers on GoaT to negotiate and split tasks to maximize the number of species sequenced, and to remove species from target lists by filtering to show those meeting EBP reference genome standards (https://www.earthbiogenome.org/report-on-assembly-standards).

Ethical Collecting

It is paramount that specimens and projects contributing to the EBP are both legally and ethically obtained. Sample collectors should ensure that all local and national permissions for collection are in place, and that there is a record of these permissions that can be referred to if any questions arise as to whether a specimen was legally obtained. This guidance applies to all species, not just those that are of conservation concern. These permissions will vary widely between countries and jurisdictions within them (e.g. [3,4] for ERGA, European Reference Genome Atlas), so it is beyond the scope of this document to summarize them. Best practice is to ensure that every specimen is collected legally within the applicable frameworks including national and local rules, rules on endangered species, rules on collecting in protected sites, and rules regarding traditional knowledge. It is also important that permissions are obtained for sequencing, shipping, and publication.

Another complicating factor is that at this phase in the project, some specimens will likely be collected and moved out of their country of origin for sequencing. Customs and import of biological material are often slow and there is a risk of losing precious material due to a loss in maintenance of the cold chain. To avoid this, proper legal documentation for export and import and associated metadata should accompany the specimens when shipped. This

can be complicated and factors to consider are described below. When shipping using a courier for the first time, it is advisable to choose a courier (e.g. World Courier and BioCair) that offers dry ice top-up or dry shipper service for a fee. Tissues that do not remain frozen for their entire journey will not yield HMW DNA or high-quality RNA unless they are in a suitable preservative. Furthermore, unexpected delays due to non-compliance with both export and import regulations are not uncommon when navigating shipments to a new country for the first time.

Regulatory paperwork could include, among others, phytosanitary and veterinary certificates that are usually obtained by country-specific legal entities (e.g. Ministries/Departments of Agriculture, National Wildlife Protection authorities, etc.), as well as other types of import and export permits allowing shipment of biological materials across the country borders. Species listed in the Convention on International Trade in Endangered Species of Wild Fauna and Flora (CITES; https://cites.org/) possess a different set of challenges, as extensive paperwork is required to obtain CITES-specific import and export permits, on top of the usual permits for shipment of biological material. CITES permits must be separately applied for from the local government authority both by the institution in the country of origin, as well as the recipient institution in the country of destination. If both institutions hold CITES Exempt Permits, this greatly simplifies the paperwork exercise. Usually, natural history museums hold these permits, and one might investigate the possibility to import/export the material of interest via those entities. If that option is not available, it is important to file all necessary paperwork with the relevant authority in both countries in advance of the intended shipping date, as it can take several months to put in order. When sending the CITES-registered species, all the necessary paperwork and customs clearing information will need to be provided to the courier company. BioCair can provide expert services in this regard, whereas many other major shipping companies might need additional guidance. It is also important to point out that some nations only allow export of biological material in special circumstances (e.g. India and China), and Brazil has a very specific set of regulations that should be checked in advance (https://www.gov.br/mma/pt-br/assuntos/bioeconomia/patrimonio-genetico/conselho-de-gestao-do-patrimonio-genetico-cgen-1/camaras-tematicas-e-

setoriais/camaras-setoriais-1/camara-setorial-da-academia/csa-faq-collections_08_01_2021.pdf).

Beyond the challenges of navigating shipments, the Nagoya Protocol (http://www.cbd.int/cop10/) and/or Access and Benefit Sharing (ABS) policies must also be followed when specimens (genetic resources) leave their country of origin. Precise guidance on following the Nagoya Protocol is beyond the scope of this document, especially given that many countries interpret the protocol differently. At a broad level, sample collectors who will be shipping specimens outside their country of origin should contact their local ABS Clearing-house (https://www.cbd.int/abs/) to understand the rules, and to obtain a PIC (Prior Informed Consent) document and a MAT (Mutually Agreed Terms on what the benefit is: financial or academic, an acknowledgement on a paper, sharing results, etc). These documents should be written as broadly as possible to support the project's vision. Countries receiving samples should ensure they have further permissions within the MAT to pass the samples on if there is any anticipation that might be required, for example to support biobanking and morphological vouchering and how data may be shared.

Beyond the rules and regulations, collecting methods must be ethical. The Earth BioGenome Project (EBP) maintains a dedicated Ethical, Legal, and Social Issues (ELSI) committee, which has published detailed recommendations for responsible sample collection, data sharing, and benefit-sharing [5]. These recommendations closely align with the Kunming-Montreal Global Biodiversity Framework (KMGBF). Adopted at the Fifteenth Conference of the Parties (COP15) to the Convention on Biological Diversity (CBD), the KMGBF represents an ambitious international commitment to halt and reverse biodiversity loss by 2030 and achieve “living in harmony with nature” by 2050. Building on earlier biodiversity targets, the KMGBF includes specific goals and targets that emphasize the need for sustainable use of biodiversity, fair and equitable benefit-sharing from genetic resources, and meaningful engagement with Indigenous Peoples and local communities [6]. These priorities resonate with the core principles of the EBP, reinforcing the project’s

commitment to inclusive and responsible biodiversity research that respects national legislation, traditional knowledge, and ABS requirements.

In practice, the KMGBF emphasizes the importance of informed consent from rights holders, equitable sharing of benefits (financial or otherwise), and capacity-building measures that support local research and conservation efforts. These provisions support the same ethical considerations championed by the EBP's ELSI committee. Consequently, EBP researchers are encouraged to familiarize themselves with the KMGBF's goals and targets to better align genomic research with current international standards for biodiversity conservation and social equity. Further considerations from the EBP's ELSI committee can be found in Sherkow et al. 2022 [5].

EBP initiatives are further encouraged to adhere to two complementary sets of data governance principles: CARE (Collective Benefit, Authority to Control, Responsibility, and Ethics) and FAIR (Findable, Accessible, Interoperable, and Reusable). The CARE principles emphasize respectful data stewardship that prioritizes the rights and interests of Indigenous Peoples and local communities [7]. The FAIR principles, meanwhile, guide the scientific community to publish genomic and biodiversity data in ways that maximize discoverability and reusability [8]. By following both CARE and FAIR, EBP researchers can ensure that ethical considerations and best practices in data management go hand in hand. Effort should also be made to build sustainable partnerships with Indigenous Peoples and local communities [3]. Overcollection of any species should be avoided. Projects should consider what the best sampling strategies might be to avoid overcollection, for example, lineage-focused bioblitzes with a group of taxonomic experts.

Confident Species Identification

Each contributed specimen should be identified to species level by a taxonomic expert and ideally, material from the same specimen should be independently DNA barcoded using appropriate markers and the data deposited publicly on the Barcode of Life Data System (BOLD; http://www.boldsystems.org) or in an INSDC database. These DNA barcodes will

ensure that species with reference genomes have independently generated DNA barcode data, that the DNA barcodes match the resulting reference genome, and that no sample swaps occur along the way. While barcoding is advised, it may not always be feasible and work is now underway by the EBP Sample Collection and Processing subcommittee to develop guidance on how to proceed with EBP goals given the challenges in confident species identification of a large proportion of Earth's biodiversity.

Robust and Complete Metadata

Robust and complete metadata of all types must accompany the family-level representatives for EBP. Metadata fields and terms should be standardized and we recommend drawing on the extensive efforts to standardize collection metadata already completed by the Biodiversity Information Standards community (originally the Taxonomic Databases Working Group, TDWG; see https://www.tdwg.org/). Both the Darwin Core standard (https://www.tdwg.org/standards/dwc/) and the Access to Biological Collection Data (ABCD) Schema (https://www.tdwg.org/standards/abcd/) offer standardized ways of sharing biodiversity occurrences and collections data. While overall similar, Darwin Core is somewhat less complex in structure, and ABCD is able to convey information at a more granular level. The GGBN Data Standard is an extension of ABCD particularly for molecular samples (https://www.tdwg.org/standards/ggbn/). The Darwin Tree of Life (DToL) project has adapted standards for collections for biodiversity genomics [9], and the latest DToL guidance can be found at https://github.com/darwintreeoflife/metadata/tree/main. Other projects have already built on the foundation provided by DToL (e.g., https://github.com/ERGA-consortium/ERGA-sample-manifest) and it is important to maintain the ontology of terms used in these different projects to prevent drift whereby terms become project-specific. Therefore it is advisable to use these metadata schemas as they are or to ensure that modifications are made in consultation. If project-specific metadata differ substantially from these recommendations, other INSDC sample checklists (e.g., https://www.ebi.ac.uk/ena/browser/checklists or https://www.ncbi.nlm.nih.gov/biosample/docs/packages/) are perfectly acceptable, provided that as many metadata fields are completed as is possible and reasonable.

The EBP recommends that every specimen should have a ToLID (tree of life identification) assigned. A ToLID is a unique, easy to communicate identifier that provides species recognition, numerically and uniquely differentiates between specimens of the same species, and adds some taxonomic context. ToLIDs facilitate internal and external communication about the samples and help the EBP track all sequencing projects. It is also worth remembering that a specimen can contain multiple organisms so ToLID can disambiguate between target and off target organisms within a given sample/specimen allowing other specimen products to be published unambiguously. Further, when a genome assembly is submitted, the ToLID of the specimen that was used for long read data generation should be used to name the assembly. ToLIDs are not a replacement for INSDC BioSample records, which hold all of the metadata associated with the sample. Every sample should have both. ToLIDs can be requested at https://id.tol.sanger.ac.uk/ (instructions on the website).

To facilitate tracking of sequencing status at the species level, and retrieving biodiversity genomics data via stable taxon identifiers, the EBP recommends assigning taxon identifiers (taxids) in the NCBI Taxonomy as early as possible in the creation of a draft target list. Taxids are stable numerical identifiers assigned by the NCBI taxonomy expert team to all taxa and taxon ranks. A species-level taxid is necessary not only for registering a ToLID for a specimen but also for declaration of intent on GoaT and for generation of BioSample and assembly submissions. Specific guidelines have been created [10] to identify the need to-, what type of- and how to- request taxids for planned sequencing targets. This starts at checking for the availability of a taxid for the target species using the ENA or NCBI Taxonomy query services. In case a taxid at species or infra-species rank for the target specimen does not exist, it needs to be requested from the ENA or NCBI Taxonomy curators as described in the guide.

Taxonomy is an active process, and revisions to species definitions, such as synonymization of existing names and splitting of existing concepts, and to the higher organisation of species into genera, families, etc., are constant. The NCBI Taxonomy aims to represent the

best supported taxonomy for all of life and strives to be up-to-date. To ensure that the NCBI Taxonomy remains current, researchers are encouraged to discuss directly with the NCBI Taxonomy curators (or the curators at ENA) whenever there is an opportunity to improve the NCBI Taxonomy, including taxonomic revisions and reconciliations.

It is important to develop a robust mechanism for updating the names and identifiers associated with a specimen if misidentification or taxonomic reclassification occurs while a reference genome is being produced. This mechanism should also have a policy to determine what gets changed at different points in the process. If a misidentification or a taxonomic name change occurs early in the process of data generation then fully updating to a new taxid, scientific name, and ToLID would be appropriate. Once the assembly has been published, we recommend correcting the taxonomic information and ToLID in any cases of misidentifications, even after release. However, we suggest that taxonomic changes like synonym preferences or merges that do not hamper the identification of the species should only be cause for amendments within six months after assembly release and the ToLID should remain as is to relieve the projects and databases from the burden of frequent minor changes.

Vouchering

Vouchers from each specimen contributing to a reference genome should be preserved when possible and guidance on various biobanking topics is available [11,12]. Ideally, there will be both morphological vouchers preserving the diagnostic characters of the taxon (or image vouchers if no material sample can be preserved), and molecular vouchers, such as tissue vouchers (discussed below), viably frozen cell lines, and/or extracted RNA and DNA. Vouchers should be prepared in a manner that ensures long-term physical preservation of the specimen and straightforward association with its metadata. Vouchers should be deposited in publicly accessible collection facilities, such as natural history museums or herbaria, located in the country of origin for each sequenced species, or where there is excess material, possibly spread across multiple repositories. It is advisable to arrange in

advance of the work commencing where remaining material (including specimen and molecular vouchers) should reside after the work is completed, and this should be reflected in any relevant legal transfer agreements. It can otherwise require secondary legal agreements to transfer materials to a third party. If remaining material is accessible, tracking additional uses of the material (e.g. by using the ToLID) is important to provide awareness to future users of the material that additional data may have already been generated on the specimen.

It is recommended that molecular vouchers be stored in a GGBN member institution for sample accessibility, and linked to the morphological voucher using a unique ID, ideally a globally unique ID (GUID, https://datatracker.ietf.org/doc/html/rfc4122), or at least following the Darwin Core triplet structure ([unique institute acronym][collection acronym, unique in the institute][voucher number, unique in the collection]). Any facility that owns and/or manages collections of non-human genomic/molecular samples can apply to become a GGBN member institute, by following the instructions here: https://wiki.ggbn.org/ggbn/Membership. When two institutions share material via an MTA (Material Transfer Agreement), it may be worth considering a specific provision that allows the remaining material to be shared with a third party if that is in the interest of the institution providing the material.

Although we strongly advocate for vouchering of specimens in publicly accessible collection facilities, we recognize that these are not uniformly available in all countries, raising potential issues with respect to export/import, access and benefit sharing, and other issues addressed in the KMGBF. Unfortunately, there is no clear solution and best practices with respect to vouchering may simply not be possible for all contributors. In such cases, we note that retention of vouchers in small institutional collections would still be preferable to not retaining vouchers at all.

High-quality images should accompany each contributed specimen, and these should be made publicly available. Although specimen digitization can involve complex photography systems, which can yield exquisitely detailed images, any photographic voucher – even one

taken with a smartphone – is better than none at all. Photographic documentation of specimens should include all relevant morphological axes. If the specimens were collected while actively engaging in identifiable behaviors (mating, feeding) these should be photographed and noted as well as the time of collection. These images can be deposited in the BioImage Archive (https://www.ebi.ac.uk/bioimage-archive/), GBIF (GBIF.org), or iDigBio (iDigBio.org). For genomes accompanied by a DNA barcode, the BOLD database may also be an option for image archiving, as well as GGBN if samples are biobanked in a member repository.

Sample to Sequence

The Phase I ambition for EBP is to sequence species that are good family-level representatives and the guidelines above indicate the primary considerations for selecting appropriate species. Ultimately, what is selected and sequenced is an individual or a set of individuals and should be recognized as so. Furthermore, additional considerations when selecting the precise specimen(s) that will be sequenced to represent the species and the family are discussed below.

Specimen and Tissue Selection

The sex of the specimen and the particular life history stages and tissues that are best for different data types should be considered carefully. Where relevant and possible, it is preferable to sample from the heterogametic sex to provide data for both sex chromosomes. Recommendations on the best life stages and best tissues to target to achieve the highest qualities and quantities of DNA, RNA, and nuclei will vary depending on the taxon. When possible, if a species has been described from a given life stage, that life stage should be selected for sequencing to decrease the likelihood of misidentification. Specific tissues might be prioritized or avoided based on additional species (cobionts) that might be present in or associated with those tissue types (e.g., sequencing gut tissue may yield off-target sequences that may or may not be desirable). The exact tissue types recommended for High Molecular Weight (HMW) DNA and RNA for the wide range of

target taxa are beyond the scope of this document. They undoubtedly will change as we experience successes and failures in extracting nucleic acids, preparing libraries, sequencing, assembly, and annotation. For annotation using RNAseq, see the EBP guidelines (https://www.earthbiogenome.org/report-on-annotation-standards). Briefly, it is recommended to collect a diversity of tissue types whenever possible, factoring in previous understanding of tissues for the focal taxa that have representative or higher-than-average transcript diversity. Over time, we will gain a better understanding of the life stages and tissues for a wide range of taxa that provide the best quantities and qualities of the material along with the most reliable protocols (e.g. for RNA extraction and for sequencing).

The target individual should be collected from the wild rather than from a laboratory colony, zoo, or culture collection (when possible), to capture natural genetic diversity, avoid inbreeding effects, and accurately reflect ecological and evolutionary contexts [13]. The size of the samples needed from a specimen will depend on the taxon and tissue sampled and the genome size, and the precise guidance around required input material is likely to be a rapidly moving target as required quantities decrease and our ability to achieve high-quality extracts across a wide range of taxa increases. Our current recommendations for animals and multicellular fungi are at least 10 and preferably closer to 100 milligrams of tissue per 1 Gb of genome size for each sample as this tends to be sufficient for long-read data generation without amplification. For plants and multicellular algae we suggest at least 100 and preferably 1000 milligrams of tissue per Gb of genome for each sample. Multiple samples from the same specimen should be prioritized over single samples from different specimens, and given the current need for samples to be directed down three different processes (Hi-C, HMW DNA extraction, RNA extraction), we recommend at least 10 samples meeting these standards per specimen where possible, and per species where not. For taxa that are known to be difficult (e.g., many marine invertebrates) and might require many extraction attempts, at least 20 samples should be taken given the significant delays and costs incurred by the need for an additional collection trip. If taking a sufficient number of samples from a single specimen is not possible, then additional specimens, ideally from the same locality and collected at the same time, should be preserved to reach

similar quantities of tissue. This level of replication gives slack in the system for repeat extractions where sufficient quantities or qualities of data have not been achieved and also provides material for biobanking, enabling future expansion of results with new approaches (e.g., protein, metabolite analysis or new or improved genome/transcriptome sequencing technologies). We strongly recommend getting in touch with the facility that is likely to complete the sequencing prior to specimen collection in the field to check their sample requirements (i.e. tube type, number of specimens required, if they accept samples preserved in ethanol or not, etc), as those vary depending on the facility and their experience in processing certain taxonomic groups.

Specimen Processing

As an organism is processed, it should be photographed alongside a tracking identifier (e.g., a SPECIMEN_ID that could be the ToLID) and alongside the barcodes of the tubes into which it is processed (Figure 3). These photographs are in addition to any that might be taken to document the living specimen and are useful for resolving sample tracking problems that can arise. We strongly encourage the use of barcoded tubes and scanning of these tubes rather than hand-writing identifiers on tubes and manual entry of identifiers into tracking systems as this is prone to error. For samples in the dozens or hundreds, this can be done with a simple single-tube scanner or even with a phone and an application like EpiCollect (https://five.epicollect.net). For larger projects processing many hundreds or thousands of specimens, rack scanners can be used to scan whole racks of barcoded tubes before sample processing.

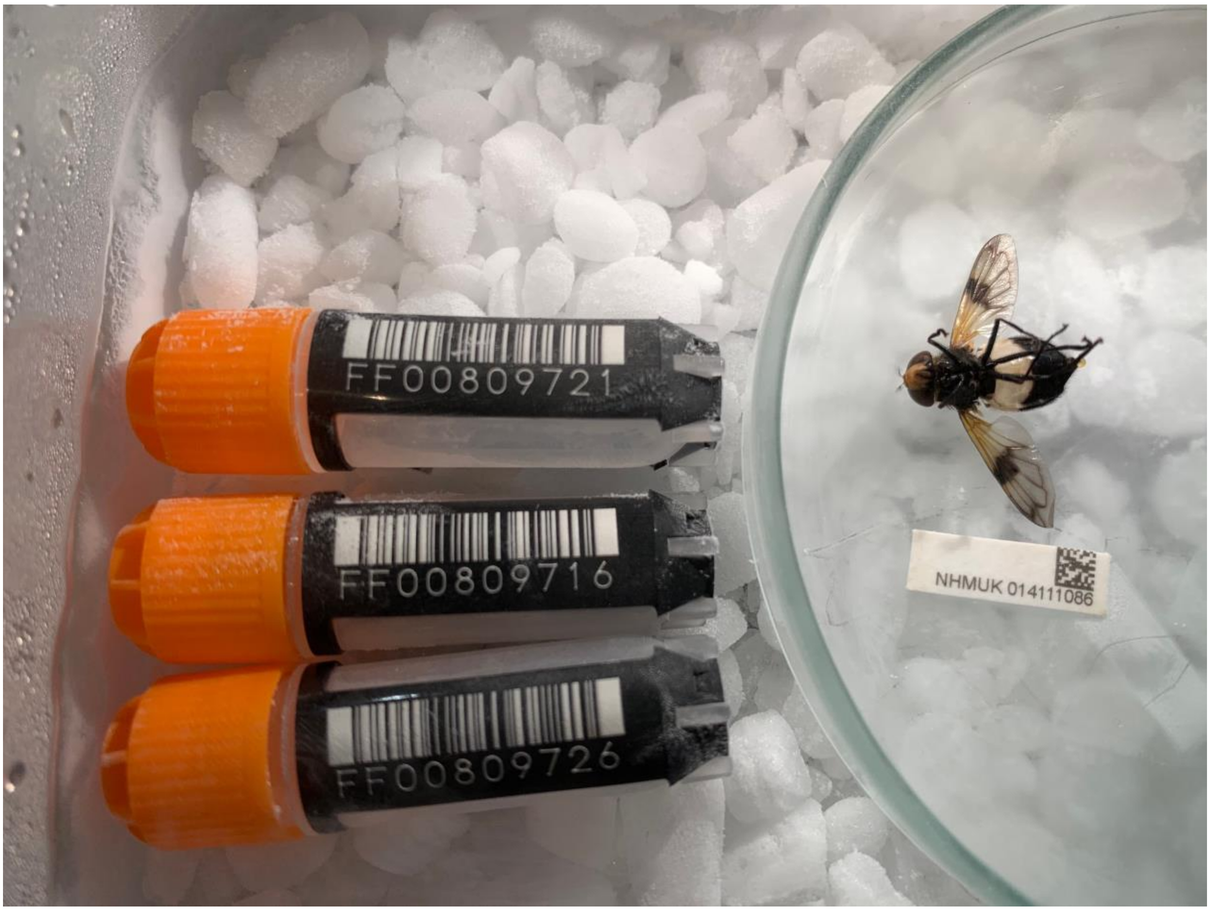

Figure 3. An example of the documentation that should occur as a sample is being processed. Here the SPECIMEN_ID (the NHM barcode under the fly) is photographed alongside the specimen and the barcoded tubes to which different samples of that specimen are destined. The metadata tracking sheet would thus have three entries for this fly, where collection-related information would be identical, but tissue type and tissue size would vary (e.g., head, thorax, and abdomen each in a separate tube). Photograph by MKNL.

Living specimens should be processed into tubes on dry ice and from that point forward, held at -70°C or below (e.g. in liquid nitrogen). Specimens that have died before processing tend to have damaged and degraded DNA and RNA, or can become highly contaminated with bacteria. Precautions should be taken if such material is to be used for reference genome generation as even if high quality DNA can be obtained from this material, it may be largely bacterial. Specimens should be taken to a site where dry ice or liquid nitrogen are available, humanely euthanized, and rapidly processed into small lentil-sized pieces (Figure 4) while freezing, for example using a petri dish on dry ice (as pictured in Figure 3) and a scalpel.

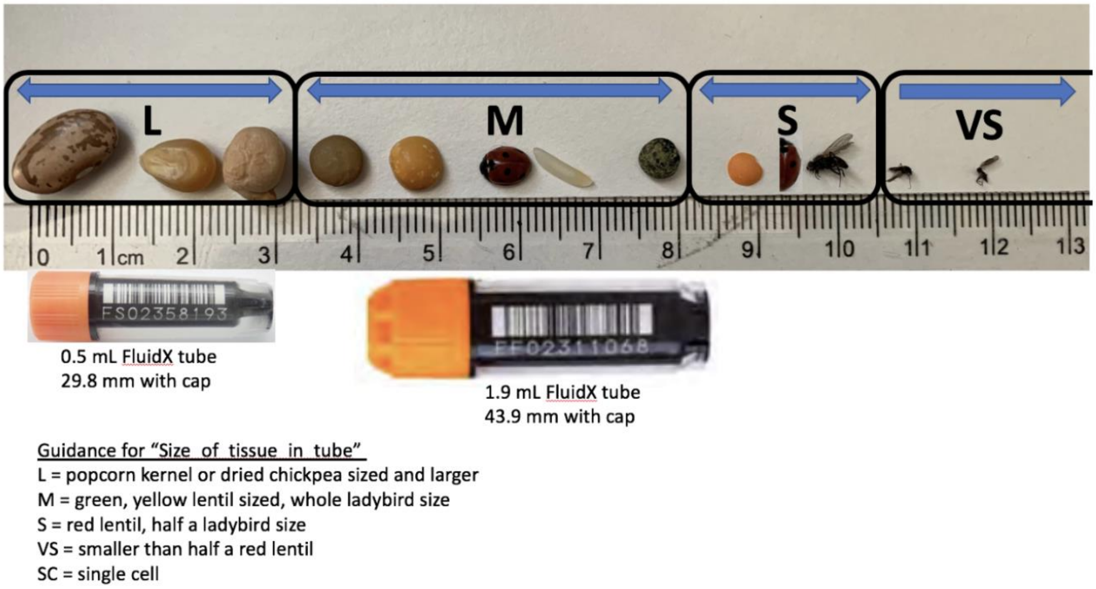

Figure 4. A scale showing the sizes of tissues that should typically be aimed for in a single tube. In some cases, (e.g. the flies in the Very Small (VS) category, there is no option to provide more tissue, so multiple separate specimens will be needed. The exact amount of tissue to supply depends on the taxon and the genome size, so it is best to discuss this in detail with the sequencing facility before beginning species collections. Facilities will not want to receive pieces of tissue much larger than the Large (L) size shown here as the tissue will be frozen solid and it is extremely difficult to break suitable sized pieces off without compromising the DNA or RNA integrity (e.g. by thawing). Plants will generally need substantially more tissue than is shown here. As advised in the main text, for each species, we recommend aiming for 10 tubes, each containing a piece of tissue in the S-L range (typically 20-50 milligrams). Photograph by MKNL.

Small pieces of specimens sitting on dry ice have generally generated high-quality DNA as long as the freezing process was rapid. These small tissue pieces can then be placed into pre-chilled barcoded tubes, e.g. in Figure 3, the fly could be cut into head, thorax, and abdomen and each piece put in a separate tube. Currently, for animals and multicellular fungi, we recommend one piece of > 10 mg tissue per tube to support different workstreams without compromising the temperature of the remainder of the material through freeze-thaw cycles. Plants and multicellular algae should also be processed into small pieces to support rapid freezing, but larger volumes of tissue might be placed in tubes as up to ten times more tissue may be required to achieve adequate quantities of DNA for these groups. For many taxa where the majority of the specimen will be consumed in the process of data generation (e.g. most insects), it may be advisable to grind the whole organism to a fine powder in liquid nitrogen prior to further laboratory work to avoid different data types being generated from different tissues (e.g., Hi-C data coming from the

head, and long-read data coming from the abdomen, each tissue with distinct associated cobiont taxa).

Sequencing the very small

For organisms where achieving milligrams of tissue is not possible including meiofauna (i.e., animals < 1 mm in size) and unicellular eukaryotes, Ultra Low Input (ULI) protocols can be adopted to generate long-read data from single microscopic organisms. Protocols leveraging long-range PCR [14,15] or multiple displacement amplification [16,17] for microscopic organisms are available with caveats that DNA integrity and genome size are important considerations and these protocols do not perform well on large, highly repetitive genomes [15,17]. We advise that single specimens be used to generate long-read data, even for the smallest organisms. A second individual may be needed to produce a transcriptome (but see [15]). Commercially available cDNA library preparation kits enable obtaining high-quality transcriptome data from a single minute animal or even single cells (e.g., [18]). Coassembly of individual single amplified genomes (SAGs) has been moderately successful for some protist species, though not yet to the standards desired by EBP [19,20]. However, pooling several individuals (ideally related individuals of the same sex/mating type) may be necessary for Hi-C. The ULI approaches might not be sufficient for achieving EBP assembly quality standards, but at this point of technology development, it is the best that can be done for many microscopic taxa. When multiple individuals must be pooled for small organisms, species and collection sites should be chosen to ideally yield several specimens; these lots need to be collected at the same time and location, and carefully determined taxonomically to ascertain whether they belong to the same species. DNA barcodes on individuals should be attempted where possible. If this is not possible for the specimens that will be used for reference genome data generation, then it is advisable to collect additional specimens from the same time and location and generate barcode data for these as a proxy identification approach. In the case of unicellular eukaryotes that are difficult to determine species, refraining from pooling cells will ensure that only a single species is sequenced. Ideally, images of the cell/organism sequenced should be taken for vouchering purposes.

Cold chain challenges

Situations in which preserving samples from living organisms without access to dry ice or liquid nitrogen are likely to become common as the EBP progresses. We are still learning which preservatives offer the best chance at successful long-read and long-range sequencing, and we recommend sharing successful protocols openly and early using the EBP protocols.io workspace (see below). As of now, we suggest if there is no possibility of rapid processing and preservation of a specimen from living to -70°C or below that samples are processed into small lentil-sized pieces in an excess (high preservative volume to tissue volume ratio) of 100% ethanol for HMW DNA and Hi-C, and in RNAlater for RNA, and that these are then stored at the coldest temperature possible. Lower percentages of ethanol are not advised as they seem to result in more degraded DNA but this is still a grey area and different taxa may have different requirements. High-quality genomes have been generated from specimens stored in lower percentages of ethanol for long periods, but here we offer general guidance on best-known practices rather than what *might* work.
For small insects that may not require further processing and so be preserved whole, compromising the cuticle to permit ethanol penetration is critical for preserving HMW DNA [21] and has resulted in high-quality reference genomes even when insects were shipped for over one week at room temperature [22]. The ratio of tissue to preservative should also be considered as water in tissues may dilute the preservative. It is advised to change ethanol twice within 24 hours following preservation for tissues with high water content. We recommend a >20:1 preservative to tissue ratio. As soon as access to a -70°C freezer is available, the samples should be frozen and records should be kept on how long samples were held at room temperature. For dissected vertebrate tissue, if liquid nitrogen is not readily available, storage in a preservation liquid for up to one week at 4°C before flash-freezing in the lab has resulted in high quality HMW-DNA and Hi-C [23].
Experience has shown that preservation in media designed for nucleic acid protection (such as DNAGuard or RNALater) is not optimal for subsequent Hi-C sequencing, as they induce excessive trans interactions, likely because of disruption of nuclei [23]. For preservation of RNA, ethanol is not suitable. RNAlater-ICE does not produce a precipitate upon cooling and

may be better than standard RNAlater for storage of RNA preserved material at below-freezing temperatures. Intensive further testing of preservatives and their ability to protect HMW DNA, RNA, and material suitable for Hi-C across the tree of life is likely to be an active area of development over the coming years, and sharing successful preservation protocols will be valuable for EBP ambitions (see below). While this summarises our current understanding of preservation when cold chain access is limited, best practice guidance is to snap-freeze tissue immediately from ethically euthanised specimens and to maintain that cold-chain until the point of extraction. This is superior in terms of HMW DNA yield and fragment size and is possible even in remote field settings with the use of charged dry shippers that can maintain ultra cold temperatures for a couple weeks.

An EBP protocols.io community

Collection, extraction, and library generation protocols are as important to retain and share, according to the FAIR principles [8], as the sample-associated metadata. We recommend sharing collection, preservation, and extraction protocols in open-source repositories where a unique Digital Object Identifier (DOI) is assigned to every document. We advise including those DOIs in publications arising from the genomes produced under the EBP umbrella. As our knowledge about the biochemical and genetic makeup of previously understudied taxa is increasing, so will the knowledge base behind the appropriate handling of samples of these taxa and their genetic material. It will increase the reproducibility of research and provide an invaluable resource for the global community.

Biologists worldwide are already actively developing and releasing protocols for ethically collecting specimens, collecting comprehensive metadata, vouchering samples, preserving and processing samples, extracting RNA and HMW DNA, sequencing RNA and DNA, performing Hi-C, etc. These protocols are abundant but still primarily focused on a small number of taxonomic groups and are also typically hidden in research manuscripts rather than released as step-by-step protocols. As we learn what modifications or entirely different approaches work best for different taxonomic groups, we encourage open sharing of this information as early as possible in protocol form. To assist with this, we have

created an Earth BioGenome Project protocols.io workspace at https://www.protocols.io/workspaces/earth-biogenome-project. We recommend that the entire community publish their genome-relevant protocols at protocols.io (this is free and results in a citable DOI) and then link their protocols to the EBP workspace. To do this, users must join the EBP workspace and once their protocol is published, simply link it as shown in Figure 5.

In addition to contributing new protocols to the EBP workspace, we also recommend extensive commenting and forking of existing protocols. Commenting on existing protocols can help rapidly share information on successes and failures, as well as minor tips and recommendations that improve chances of success. Commenting and forking are easily achieved on any protocol through the "COMMENTS" and "COPY / FORK" clickable buttons that are present on every protocol. We suggest using comments to indicate if the protocol worked for specific species and to include higher taxonomic information, e.g. "This protocol results in high-quality HMW DNA for *Hapalochlaena lunulata*, an octopus. The DNA was successfully sequenced using PacBio HiFi". Forking can be used where an existing protocol has been modified more extensively to improve results, for example for a particular tissue or taxon.

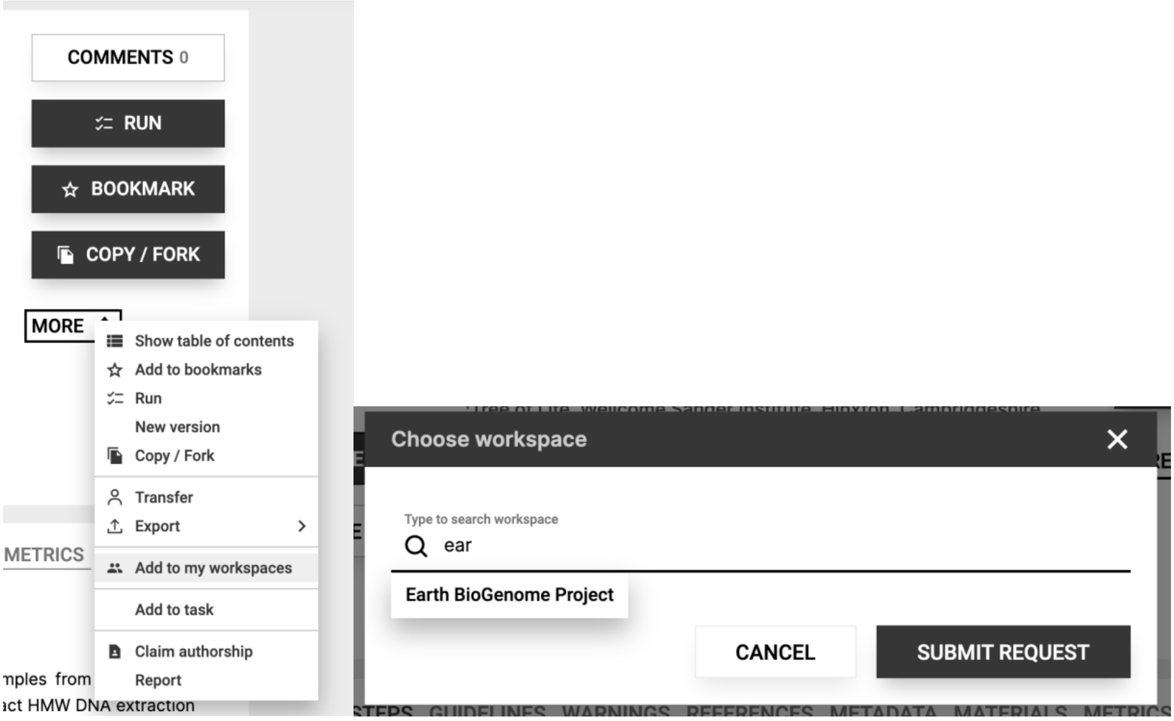

Figure 5. How to link protocols to the EBP workspace. Step 1. Join the Earth BioGenome Project Workspace at https://www.protocols.io/workspaces/earth-biogenome-project Step2. Click on the "MORE" menu as shown in the left panel above and select "Add to my workspaces". Step 3. Select "Earth BioGenome Project" as the workspace and add the protocol.

In the process of trying to go from specimen to high quality reference genome, we learn through unexpected successes and failures the tips and tricks that are not protocol-worthy, but still should be shared. If we had a way to share these anecdotes, we might save people treading down the same dead end roads or give them a tip that opens up huge possibilities. Therefore, we have created a community owned Google sheet called "EBP community collection of anecdotes" (https://tinyurl.com/EBP-Anecdotes). This is set up to simply collect experiences of anyone working the general area of reference genomes for biodiversity. We ask that the EBP community continue to populate this resource with their tips and tricks and we have provided some guidelines at the top of the document.

Fostering Inclusive Collaboration for Enhanced Impact

For the Earth BioGenome Project to succeed, we must all work towards a more inclusive and equitable global biodiversity genomics community. Strengthening collaboration and engagement across the biodiversity genomics stakeholder community will accelerate progress and maximize scientific impact, benefit sharing, equity, diversity, inclusion, and just practices. The biodiversity genomics stakeholder community includes individuals from all relevant subdisciplines of biology: those traditionally engaged, such as geneticists, genomicists, and bioinformaticians; experts in taxonomy, natural history and ecological and evolutionary theory, including ecologists, evolutionary biologists, taxonomists, and systematists; and those often overlooked, such as individuals working in natural history collections who are involved in vouchering and specimen curation, staff at sequencing facilities who assist with project design and data generation, and those providing support in other aspects of project design, specimen acquisition, data generation, and curation.

The success of EBP is intrinsically linked to the collaborative efforts of scientists across a wide range of disciplines. Researchers working in natural history collections, at biobanks, taxonomists, systematists, genomicists, bioinformaticians, and support staff at sequencing facilities all play crucial roles. These professionals collect, identify, preserve, and maintain specimens, infer phylogenetic relationships, and study the evolution of traits and geographic distributions of organisms. Their work enables the genomic research community to access well-curated and accurately identified specimens, ensuring the correct identification and systematic placement of species-representative reference genomes. It is essential for all involved to acknowledge and strengthen these relationships, advocating for the vital work done by museum scientists, systematists, taxonomists, and technical support staff. Additionally, relevant taxonomic and systematic work, such as species descriptions and evolutionary context studies, and IDs of collection material examined, should be cited in genomics publications. Ensuring that individuals who collected and identified specimens are included as authors on resulting papers fosters a collaborative approach and gives credit where it is due [24].

Enhanced collaborations, e.g., among genomicists, systematists, taxonomists, museum scientists, bioinformaticians, and sequencing facility staff improve the scientific rigor of genomic research and recognize the critical contributions of experts in taxonomy, evolution and organismal biology [25,26]. Systematics and taxonomy provide an indispensable framework for genomics research. Experts in these fields often lead advances in imaging, informatics, ecology, evolution, genetics, and genomics (e.g., [27–29]), and often yield important new discoveries at the interface between traditional subdisciplines of biology (e.g., [30,31]). Presently, many groups of organisms are studied by only a few living taxonomic experts, making reliable identification challenging, at best. Alarmingly, limited taxonomic training in undergraduate curricula, fewer living expert taxonomists mentoring students and postdocs, and limited funding for taxonomic research stand to exacerbate this issue [26]. Collaborative efforts can provide valuable training for the next generation of integrative biodiversity scientists.

Close collaborations among genomicists, museum scientists and taxonomists can also prevent duplicative sampling efforts, minimize the ecological impact of specimen collection, conserve resources, and ensure efficient sampling. With genomic sequencing now more cost-effective, it is crucial to maximize the value of each collected specimen [32]. The genomics community, with its expertise in bioinformatics and data management, can significantly contribute to biodiversity informatics and digitization initiatives [33]. Researchers collaborating with museums can advocate for best practices in specimen preservation that will facilitate future genomic work and ensure the deposition of voucher specimens in public collections [34]. Of course, many scientists in the field of biodiversity genomics also engage in systematics and taxonomy and/or work in museum settings, demonstrating the interdisciplinary nature of modern genomics research (e.g., [35]).

All members of the EBP stakeholder community, including genomicists, systematists, taxonomists, museum scientists, bioinformaticians, sequencing facility staff, and others, play important interdependent roles in this endeavor. Strengthening collaboration among these communities will enhance the quality and impact of research, ensuring it is ethical, sustainable, and inclusive. Through such partnerships we can more fully and effectively explore and preserve the diversity of life on Earth for generations to come.

Looking to the Future

Current best practice assembly guidelines are to generate a combination of data types including long-read (PacBio HiFi and/or ultra-long ONT), long-range (Hi-C), and RNAseq (Illumina short read, PacBio Kinnex, or ONT cDNA-PCR) data from the same specimen wherever possible, aiming for the heterogametic sex when this is relevant. Typically, separate samples are used for these different applications, but we should be developing protocols that support minimal extraction of material sufficient for any of these types of data generation (e.g., nuclei co-extracted with RNA). Furthermore, in all current extraction efforts, we discard material that we might one day look back on and regret, such as proteins and metabolites. While data generation from these materials is currently out of scope, this is unlikely to be true in years to come. Retaining relevant material to add data

layers to the high-quality reference genomes would be prudent. Thus, for specimens where samples are available in excess, considerations should be given to preserving replicate samples and appropriate storage to future-proof these samples as much as possible. For specimens where all material is used in data generation, perhaps typically discarded supernatants should be retained for future investigations.

As sequencing proceeds into phases comprising more fine-scale taxonomic coverage, we foresee that getting specimens identified *in vivo* before euthanizing and freezing will be challenging for many species-rich but poorly known taxa, including most invertebrate groups. Reliable identifications often depend on careful examination under the microscope by experts, of which there are typically only a few for entire taxonomic families; this would require holding the specimens out of the cold chain for a length of time that could compromise the DNA and RNA. This highlights the importance of appropriately preserved morphological voucher specimens and imaging. Further, advances in DNA barcoding reference libraries can be of enormous importance, so that specimens can be collected first and identified later. One possibility would be to have a fourth tissue sampling used initially only for DNA barcoding to attempt identification by comparing the sequence to available public databases. Such identifications would then inform the decisions about whether a sample should be further processed for genomic sequencing.

We also encourage activities that simplify and streamline standard operating procedures and protocols to make it easier to “containerize” extraction, sequencing, and assembling activities. Containerization means a world in which a simple portable lab could house everything needed to go from sample to sequence and would build capacity in the Global South, in the nations that often harbor the greatest biodiversity. This relieves pressure on Nagoya requirements and the budget spent on expensive shipping costs to maintain the cold-chain, but more importantly, it is better for global science.

Numerous unexplored opportunities also lie within the realm of AI and automation, waiting to be unlocked and harnessed. AI-driven robotic systems could increase efficiency by automating sample collection and optimizing workflows, significantly reducing the time and

resources required (e.g. [36]). This integration could enhance precision and accuracy through AI-based quality control and precise data annotation, ensuring only high-quality samples contribute to genome sequencing. Costs could thus be reduced through labor savings and resource optimization, while the speed of genome sequencing could be accelerated by automated processing and parallelization of samples. Furthermore, AI could be used to facilitate intelligent sampling strategies, promoting diversity in collected samples, and enabling real-time monitoring of environmental conditions. Integrating biodiversity data into centralized databases and advanced analytics enhances data accessibility and analysis. Overall, the synergy of AI and automation may improve the quality and speed of reference genome generation. Reference genomes from across the tree of life enabled by a global community and multiple technologies will contribute to a deeper understanding of biodiversity for applications in conservation, ecology, and evolutionary biology.

Abbreviations

EBP = Earth BioGenome Project

GoaT = Genomes on a Tree

Gb = Gigabase

INSDC = International Nucleotide Sequence Database Collection

ABS = Access and Benefit Sharing

BOLD = Barcode of Life Database

ToLID = Tree of Life Identification

Taxid = Taxonomic identification

MTA = Material Transfer Agreement

HMW = High Molecular Weight

PacBio = Pacific Biosciences

ONT = Oxford Nanopore Technologies

Declarations

Data Availability: No data was generated or used in the drafting of this article.

Competing Interests: The authors declare that they have no competing interests.

Funding: Authors MKNL, MB, and CGSC are funded by the Wellcome Trust quinquennial award to the Wellcome Sanger Institute (grant number 220540/Z/20/A). OVP is funded by the SciLifeLab (Science for Life Laboratory, Sweden), and the Swedish Research Council (RFI/VR). KMK is funded by National Science Foundation grants 1846174, 2138994, 2321308, and 2001303. VR is

funded by the NIH Common Fund Award / NHGRI Grant Number U24HG006941 and National Institutes of Health (OD), National Institution of Biomedical Imaging and Bioengineering, and NIH award number 1 U2C EB 032224 - 01. DDM is funded by National Science Foundation grants 2110053 and 1937815. JGW is funded by the National Science Foundation grant DBI-2119963, BII: Mechanisms of Cellular Evolution. AKC is supported by the U.S. Department of Agriculture, Agricultural Research Service (USDA-ARS), Bee Research Laboratory in-house appropriated research project 8042-21000-291-000-D.

Authors' Contributions: The EBP Sample Collection and Processing sub-Committee members conceptualized the manuscript. M.K.N.L. drafted the original manuscript. All authors contributed to review and editing. EBP Sample Collection and Processing sub-Committee as of 2025: Chair Mara K. N. Lawniczak and all members (listed alphabetically): Jonas Astrin, Anna Childers, Kevin Kocot, Duane McKenna, Olga Pettersson, Verena Ras, Bernardo Santos, and Jeremy Wideman.

Acknowledgements: The authors thank Harris Lewin and Federica di Palma for comments.

References

1. Blaxter M, Archibald JM, Childers AK, Coddington JA, Crandall KA, Di Palma F, et al. Why sequence all eukaryotes? Proc Natl Acad Sci U S A. 2022;119. doi:10.1073/pnas.2115636118

2. Challis R, Kumar S, Sotero-Caio C, Brown M, Blaxter M. Genomes on a Tree (GoaT): A versatile, scalable search engine for genomic and sequencing project metadata across the eukaryotic tree of life. Wellcome Open Res. 2023;8: 24. doi:10.12688/wellcomeopenres.18658.1

3. Mc Cartney AM, Head MA, Tsosie KS, Sterner B, Glass JR, Paez S, et al. Indigenous peoples and local communities as partners in the sequencing of global eukaryotic biodiversity. NPJ Biodivers. 2023;2: 8. doi:10.1038/s44185-023-00013-7

4. Böhne A, Fernández R, Leonard JA, McCartney AM, McTaggart S, Melo-Ferreira J, et al. Contextualising samples: supporting reference genomes of European biodiversity through sample and associated metadata collection. NPJ Biodivers. 2024;3: 26. doi:10.1038/s44185-024-00053-7

5. Sherkow JS, Barker KB, Braverman I, Cook-Deegan R, Durbin R, Easter CL, et al. Ethical, legal, and social issues in the Earth BioGenome Project. Proc Natl Acad Sci U S A. 2022;119. doi:10.1073/pnas.2115859119

6. COP15: Final text of Kunming-Montreal Global Biodiversity Framework. In: Convention on Biological Diversity [Internet]. [cited 28 Jan 2025]. Available: https://www.cbd.int/article/cop15-final-text-kunming-montreal-gbf-221222

7. Carroll SR, Garba I, Figueroa-Rodríguez OL, Holbrook J, Lovett R, Materechera S, et al. The CARE principles for indigenous data governance. Data Sci J. 2020;19. doi:10.5334/dsj-2020-043

8. Wilkinson MD, Dumontier M, Aalbersberg IJJ, Appleton G, Axton M, Baak A, et al. The FAIR Guiding Principles for scientific data management and stewardship. Sci Data. 2016;3: 160018. doi:10.1038/sdata.2016.18

9. Lawniczak MKN, Davey RP, Rajan J, Pereira-da-Conceicoa LL, Kilias E, Hollingsworth PM, et al. Specimen and sample metadata standards for biodiversity genomics: a proposal from the Darwin Tree of Life project. Wellcome Open Res. 2022;7: 187. doi:10.12688/wellcomeopenres.17605.1

10. Blaxter M, Pauperio J, Schoch C, Howe K. Taxonomy Identifiers (TaxId) for Biodiversity Genomics: a guide to getting TaxId for submission of data to public databases. 2024 [cited 15 Oct 2024]. doi:10.12688/wellcomeopenres.22949.1

11. Corrales C, Astrin JJ. Biodiversity Biobanking – a Handbook on Protocols and Practices. AB. 2023;1: Advanced Books. doi:10.3897/ab.e101876

12. ISBER. [cited 21 May 2024]. Available: https://www.isber.org/page/BPR

13. Frankham R. Genetic adaptation to captivity in species conservation programs. Mol Ecol. 2008;17: 325–333. doi:10.1111/j.1365-294X.2007.03399.x

14. Schneider C, Woehle C, Greve C, D'Haese CA, Wolf M, Hiller M, et al. Two high-quality de novo genomes from single ethanol-preserved specimens of tiny metazoans (Collembola). Gigascience. 2021;10. doi:10.1093/gigascience/giab035

15. Laumer C. Picogram input multimodal sequencing (PiMmS). 2023. Available: https://www.protocols.io/view/picogram-input-multimodal-sequencing-pimms-rm7vzywy5lx1/v1

16. Lee Y-C, Ke H-M, Liu Y-C, Lee H-H, Wang M-C, Tseng Y-C, et al. Single-worm long-read sequencing reveals genome diversity in free-living nematodes. Nucleic Acids Res. 2023;51: 8035–8047. doi:10.1093/nar/gkad647

17. Roberts NG, Gilmore MJ, Struck TH, Kocot KM. Multiple Displacement Amplification Facilitates SMRT Sequencing of Microscopic Animals and the Genome of the Gastrotrich Lepidodermella squamata (Dujardin, 1841). bioRxiv. 2024. p. 2024.01.17.576123. doi:10.1101/2024.01.17.576123

18. Varney RM, Funch P, Kocot KM, Sørensen MV. A new species of Echinoderes (Cyclorhagida: Echinoderidae) from the San Juan Islands, Washington State, USA, and insights into the kinorhynch transcriptome. Zool Anz. 2019;282: 52–63. doi:10.1016/j.jcz.2019.06.003

19. Mangot J-F, Logares R, Sánchez P, Latorre F, Seeleuthner Y, Mondy S, et al. Accessing the genomic information of unculturable oceanic picoeukaryotes by combining multiple single cells. Sci Rep. 2017;7: 41498. doi:10.1038/srep41498

20. Sørensen MES, Zlatogursky VV, Onuţ-Brännström I, Walraven A, Foster RA, Burki F. A novel kleptoplastidic symbiosis revealed in the marine centrohelid Meringosphaera with evidence of genetic integration. Curr Biol. 2023;33: 3571–3584.e6. doi:10.1016/j.cub.2023.07.017

21. Teltscher F, Lawniczak M. Squishing insects for preservation of HMW DNA in the field. 2023 [cited 24 Jan 2024]. Available: https://www.protocols.io/view/squishing-insects-for-preservation-of-hmw-dna-in-t-4r3l2224jl1y/v1

22. Nsango SN, Ayala D, Agbor J-P, Johnson HF, Heaton H, Wagah MG, et al. A chromosomal reference genome sequence for the malaria mosquito, *Anopheles nili*, Theobald, 1904. 2024 [cited 16 Oct 2024]. doi:10.12688/wellcomeopenres.23198.1

23. Dahn HA, Mountcastle J, Balacco J, Winkler S, Bista I, Schmitt AD, et al. Benchmarking ultra-high molecular weight DNA preservation methods for long-read and long-range sequencing. Gigascience. 2022;11: giac068. doi:10.1093/gigascience/giac068

24. Meier R. Citation of taxonomic publications: the why, when, what and what not:

Species citations. Syst Entomol. 2016;42. doi:10.1111/syen.12215

25. Drew LW. Are We Losing the Science of Taxonomy? As need grows, numbers and training are failing to keep up. Bioscience. 2011;61: 942–946. doi:10.1525/bio.2011.61.12.4

26. Löbl I, Klausnitzer B, Hartmann M, Krell F-T. The Silent Extinction of Species and Taxonomists—An Appeal to Science Policymakers and Legislators. Diversity . 2023;15: 1053. doi:10.3390/d15101053

27. Faulwetter S, Vasileiadou A, Kouratoras M, Thanos Dailianis, Arvanitidis C. Micro-computed tomography: Introducing new dimensions to taxonomy. Zookeys. 2013; 1–45. doi:10.3897/zookeys.263.4261

28. Meyer C, Duffy E, Collins A, Paulay G, Wetzer R. The U.S. Ocean Biocode. Mar Technol Soc J. 2021;55: 140–141. doi:10.4031/MTSJ.55.3.33

29. Pante E, Schoelinck C, Puillandre N. From integrative taxonomy to species description: one step beyond. Syst Biol. 2015;64: 152–160. doi:10.1093/sysbio/syu083

30. McKenna DD, Shin S, Ahrens D, Balke M, Beza-Beza C, Clarke DJ, et al. The evolution and genomic basis of beetle diversity. Proc Natl Acad Sci U S A. 2019;116: 24729–24737. doi:10.1073/pnas.1909655116

31. Stiller J, Feng S, Chowdhury A-A, Rivas-González I, Duchêne DA, Fang Q, et al. Complexity of avian evolution revealed by family-level genomes. Nature. 2024;629: 851–860. doi:10.1038/s41586-024-07323-1

32. Funk VA. Collections-based science in the 21st Century. J Syst Evol. 2018;56: 175–193. doi:10.1111/jse.12315

33. Guralnick R, Hill A. Biodiversity informatics: automated approaches for documenting global biodiversity patterns and processes. Bioinformatics. 2009;25: 421–428. doi:10.1093/bioinformatics/btn659

34. Colella JP, Stephens RB, Campbell ML, Kohli BA, Parsons DJ, Mclean BS. The Open-Specimen Movement. Bioscience. 2021;71: 405–414. doi:10.1093/biosci/biaa146

35. Droege G, Barker K, Astrin JJ, Bartels P, Butler C, Cantrill D, et al. The Global Genome Biodiversity Network (GGBN) Data Portal. Nucleic Acids Res. 2014;42: D607–12. doi:10.1093/nar/gkt928

36. Wührl L, Pylatiuk C, Giersch M, Lapp F, von Rintelen T, Balke M, et al. DiversityScanner: Robotic handling of small invertebrates with machine learning methods. Mol Ecol Resour. 2022;22: 1626–1638. doi:10.1111/1755-0998.13567

Personal Cover Click here to access/download;Personal Cover;Response to reviewers.docx

Dear Editor
We have addressed the reviewers comments and responded below. Thank you for considering this manuscript.

Reviewer #1: Review of Lawniczak et al. Best Practice Guidance for Earth BioGenome Project Sample Collection and Processing: Progress and Challenges in Biodiverse Reference Genome Creation.

The important manuscript is very well-written and organized, and the authors are to be complemented on this. The manuscript offers many links to relevant websites, and in doing so it is a valuable resource.

Thank you.

The manuscript is particularly applicable to family-level collecting, but will have limited utility in the long term. For example, once the project moves to genus-level analyses, specimen acquisition will increasingly require extended fieldwork in remote areas. This activity will complicate the initial freezing of samples on dry ice as return to a laboratory or place where LN2 or dry ice is easily acquitted will be impossible. Although this is mentioned, I would suggest that the authors might want to add a bit of text relative to this eventuality.

We agree that guidance and best practice will change over time and this EBP subcommittee's tasks will be to maintain this document and update guidance periodically. It is possible that GIGASCIENCE will also accept future updated versions.

I am surprised that the manuscript does not mention CITES other than "It is also important that permissions are obtained for... shipping ..." Shipping of CITES I species is quite complicated in requiring both export and import permits. One means to facilitate such shipments involves using CITES Exempt Permits held by museum, and rarely other institutions. Perhaps the authors should expand a bit on shipping constraints. Another issue, of course, is that some countries to not allow the exportation of bioresources (e.g., India).

Indeed shipping of CITES species requires that both the exporting individual and the receiving individual has to be registered with CITES, and such registration and import administration is controlled by official bodies in each nation. We have added text on this topic.

Re. "Researchers are encouraged to discuss directly with the NCBI Taxonomy curators (or the curators at ENA) [note space typo] whenever there is an opportunity to improve the NCBI Taxonomy, including taxonomic revisions and reconciliations." Unfortunately, for species identifications, changes can only be made by the submitter of the data, and once an error is created, publications usually perpetuate it. Thus, I believe the text might be better-phrased to reflect current practices at NCBI.

It is true that the species allocation of a sequence can only be changed by the submitter, but the text refers to the treatment of species (and subspecies, strains, etc) in the hierarchy of TaxonomyDB. We have adjusted the text to make this clearer. The NCBI Taxonomy DB curators are fully able to change the taxonomy *displayed* for a sequence, while keeping the taxonomy as submitted as an archival statement in the database. Sequences are deposited attached to a TAXID, a numeric value. This TAXID points to the standard species name, and the position of the species in the wider taxonomy is inferred by the taxonomic tree within TaxonomyDB. Thus if two species are later synonymised, this change is effected "behind the scenes" by changing the linkage of the TAXIDs. The TaxonomyDB curators actively engage with the taxonomic community to improve their representation of wider taxonomy, especially as major revisions are carried out. The curators work carefully and assiduously to ensure that the representation of taxonomy is as correct as possible, but do not aim to be cutting edge or radical, and are inherently conservative in their requests for change.

Re. "It is also worth bearing in mind that if you are sharing material with another institute and an MTA (material transfer agreement) is in place, make sure that the MTA states that remaining material can be shared with a third party if you so wish." I disagree with this as the voucher-holder must be the sole controller of resource distribution. There's an infamous case of looking at genetic variability in a species based on a few samples. No variability was found. As it turned out, all independent sequencing was done using the same sample, which had been distributed to several labs independently. At least all sequencing of the same individual was repeatable!

This is an interesting point and we have added in a sentence to ensure care is taken in sharing material for additional sequencing, particularly paying attention to sample metadata and unique identifiers.

Reviewer #2: This manuscript is an effort to "outline recommended standards and considerations in sample acquisition and processing" for the Earth Biogenome Project (EBP).

The paper's sections include background, recommendations for sampling, openly collating genome sequencing proposals and efforts underway, ethical collecting,

confident species identification, robust and complete metadata, vouchering, selection and processing of samples, establishment of a community io for protocols, fostering inclusive collaboration, and consideration and recommendation of future modifications or improvements relevant to sample acquisition and processing.

Addressing crucial aspects of the EBP involves consideration of diverse disciplines and technologies, requiring informed input from multiple authors. It appears that the sections of the paper may have been distributed to specific authors, this deduced from the variation in style and voice of the sections. All the sections address significant aspects of a comprehensive presentation and discussion of collection and processing of useful samples for generation of annotated reference genome assemblies across the Tree of Life. However, they are uneven in their scope and depth.

A major revision of the manuscript can re-organize the inclusion of topics so that they appear together in logical order. The depth of the sections could be more uniform. Each section could state which constituents of the broad EBP community the section addresses, and if all, so stated. It will be helpful to make it as much like a manual as feasible. Reference to common elements among and between sections would also be helpful.

This paper aims to bring awareness to the factors that should be considered when collecting, shipping, and processing specimens for reference genomes and to offer high level best practice suggestions. It also aims to bring awareness of a wide range of topics to each EBP community member contributing to the project, irrespective of whether they are contributing to the collection, vouchering, identification, or otherwise. Within the manuscript, we also promote the idea that specific Standard Operating Procedures (e.g. manuals of the sort you might be looking for) will continue to be developed and published at the EBP protocols.io Workspace but guidance at the detailed level as would be required for manuals is beyond what we think is appropriate for a manuscript.

As Reviewer 2 notes, the flow of the current layout is

- Recommendations for success focused on phase 1 - a species for every taxonomic family.
- EBP phase 1 target species collectively displayed by each contributing project via GOAT.
- Ethical collecting
- Confident species ID
- Robust and complete metadata
- Vouchering
- Sample to sequence (including choosing specimens & tissues, processing specimens, and extra challenges from the very small & the need for cold chain when shipping is required)

- Then we promote the EBP protocols.io workspace
- Our main text ends with the key section on fostering inclusive impact, as this is how the EBP goals will be met.

We do not see an obvious structural problem with this layout – this was the logical order to us. The reviewer does not suggest a different order apart from moving the fostering impact section to the beginning, which we disagree with as we think that the reader will remember this better if it is at the end of the manuscript after having read more of the practical details described above. We also think that by stating “which constituents of the broad EBP community each section addresses” that we risk oversimplifying – having every section read by every EBP community member means that collectors have a better understanding of downstream processes, and museum scientists have a better understanding of the necessary best practices across the full chain. This isn’t meant to be a manual that you could turn to page x and read the relevant section – it is meant to offer a holistic view on best practice on all factors that one should consider when setting out to do sample collection for reference genomes.

For example, discussions of vouchering appear in several sections and are at times contradictory, i.e., vouchers or not; taxonomic identification or not. In some sections the tone is directive ("must") and in places is less so ("recommended"). For an international, diverse audience the tone of the prescriptions should be carefully explained and rationalized.

We believe that the contradictory messages the reviewer has identified appear because of the distinction between more straightforward (e.g. large specimens, identifiable to species) and less straightforward (e.g. microscopic, not identifiable to species) specimens. We have revised the manuscript to be clearer on this distinction. We have also removed every instance of “must” apart from those around ethics where a strong stance on ethical collecting and following ABS procedures is important because this manuscript is written on behalf of EBP.

Overall, the treatment of the ethical issues is less than what should be expected. The ultimate success of the EBP will be strongly influenced by perceptions across cultures, tribes and nationalities, and the signatories to the Convention on Biological Diversity (CBD). In the section on ethical collecting the Kunming-Montreal Global Biodiversity Framework (KMGBF) is not mentioned. Similarly, the adherence to the CARE and FAIR principles and prior informed consent is not in the manuscript.

The reviewer is correct that these are key considerations of the EBP. There is an EBP committee focused on ethical issues. The topic is broad, complex, and important enough that it warrants its own manuscript, which has already been published regarding the

ethical, legal, and social issues of the EBP (https://www.pnas.org/doi/10.1073/pnas.2115859119). This was published shortly before KMGBF goals were adopted, so we have added a short section in our manuscript on this topic specifically. We do already discuss FAIR principles and prior informed consent in the manuscript, and we have now also added CARE principles in the relevant section.

> In the discussion of Confident Species Identification, standards are called for that later in the manuscript are discussed as difficult to impossible.

This relates to the easier vs harder species and also the different phases of the project. As noted above, we have now made this clearer in the manuscript.

> In the section of Robust and Complete Metadata, there are many links in the text to complex documents, the portion of which is not clearly explained. To better make the desired points, some summary or explanation of what is desired is warranted. References to identifiers appear in this and other sections. Given the importance, perhaps a separate section about this that clearly compiles and/or references relationships among databases and sample IDs. The discussion of a robust mechanism for updating the names and identifiers brings a wealth of terms: taxids, taxonomies, ToL IDs, ENA or NCBI. Taxonomy query services, are discussed as well as timelines for reconciliation/revision. Why is the date for amendments suggested to be 6 months?

We acknowledge that by trying to capture the multitude of possible scenarios, we overcomplicated the recommendations. In order to make them easier to understand, we simplified the scenarios and added some text to describe the identifiers.

The time limit for amendments is arbitrary and based on practical experience. Whilst we recommend correcting all mistakes, however long in the past, we can't commit to apply changes to capture e.g. new synonyms that don't affect the identification of a species. We amended the text to make this clearer.

> In the section on Vouchering, recommended is that vouchers be deposited in publicly accessible collection facilities, such as natural history museums or herbaria and specifically in GGBN-member institutions. These are not uniformly available facilities in countries, thereby raising the issue of export/import, access and benefit sharing, and other issues addressed in the Kunming-Montreal Global Biodiversity Framework (KMGBF). The recommendation that high-quality images should accompany each contributed specimen, with documentation including all relevant morphological axes, similarly poses challenges to inclusion of all CBD signatories. The recommended equipment/workstations is expensive equipment, not uniformly available in all

countries, raising the issue of export/import, Nagoya Protocol, benefit sharing, and KMGBF.

We thank the reviewer for raising these points. While we maintain that, when possible, best practice dictates voucher specimens should be retained and made available to the research community through deposition in a publicly accessible collection facility/database, we agree that such facilities are not uniformly available. Unfortunately, there is no clear solution and this will simply not be possible for all contributors. This caveat to our advocated best practices has been noted in detail in a new paragraph added to the Vouchering section of the manuscript.

With respect to the reviewer's comment regarding specimen photography, we respectfully disagree that the advocated for practices pose significant challenges to inclusion of all CBD signatories. This text has been revised to better clarify our intention of advocating for taking and sharing the best-possible quality photos.

In the section on Sample to Sequence, within the discussion of Specimen and Tissue Selection, the discussion of challenges and approaches to obtaining HMW DNA across taxa identifies the need for community discussion, data sharing, and need to undertake complex and redundant sampling and storage procedures, noting that "recommendations will vary." In the section on Specimen Processing, a SPECIMEN_ID is recommended, along with a photo of the sample tubes, voucher tag, and specimen.Here it might be useful to allude to in the discussion of identifiers as part of the chain linking data to specimen and harmonize the recommendations.

We have added that SPECIMEN_ID here could be a TOLID.

The section on Sequencing the very small discusses the array of challenges that must be addressed with species of minute mass/volume and provides suggestions, while alluding to uncertainties, again raising the issue of taxonomic identification and complete destruction of the individual in order to obtain a suitable specimen. Later, the use of BARCODES, is raised and it might well appear here.

We have added that barcodes can perhaps be used on proxy individuals.

In the section on Cold chain challenges, the issue of transfer of specimens is brought up. This has been mentioned in other sections, but is an issue of significance, if transfer is across international borders, due to Nagoya and KMGBF. For this article to be useful into the future, this aspect needs more prominence and careful consideration for the success of the EBP.

We have added guidance to address the challenges of maintaining cold chain across international borders.

> The proposal for a EBP protocols.io community is a very useful suggestion, allowing for a community participation in rapid updating and communicating significant information crucial to the success of the EBP (including the EBP anecdotes).

> The section on Fostering Inclusive Collaboration for Enhanced Impact seems to me to be first principle and therefore brought in earlier in the manuscript. The EBP will fail if is not or is perceived not to be inclusive, equitable, involve all signatories to the CBD, and incorporate the CARE and FAIR principles.

We completely agree that this is a first principle but also feel that the location of this topic is more impactful at the end of the manuscript when the details of the best practice have been covered. We have added in some additional sentences to this section to reflect on how core it is to the success of the project.

> In the section on Looking to the Future, the utility of DNA barcoding of libraries to assist in specimen and sample verification is brought up. An important point and that should be brought up in the earlier discussions of samples and taxids.

DNA barcoding is covered in the confident species ID and we have now also discussed it again in the revised section on taxid.

> This section ends with speculation of the crucial significance of AI in the EBP at all stages. This is warranted, but as it is expected to be impactful in ways. not currently evident, the ending of the manuscript in this way could be strengthened by reinforcing the value to human society and planetary ecosystems that support it, and of which humankind is a part. Having reference genomes across the Tree of Life, enabled by multiple technologies, including AI "contribute[s] to a deeper understanding of biodiversity for applications in conservation, ecology, and evolutionary biology."

Thank you, we have made this revision.

> Additional comments are made on the attached PDF copy of the manuscript.

Thank you, we have addressed these other changes as requested in the PDF.
