# Supplementary material for: Best-practice guidance for Earth BioGenome Project sample collection and processing: progress and challenges in biodiverse reference genome creation
Source: Gigascience. 2025 May 29;14:giaf041. doi: 10.1093/gigascience/giaf041 (PMC12121479; doi:10.1093/gigascience/giaf041)
Supplement: giaf041_Reviewer_1_Report_original_submission — Robert Murphy -- 12/3/2024 [file giaf041_reviewer_1_report_original_submission.pdf]

Reviewer Report

Title: Best Practice Guidance for Earth BioGenome Project Sample Collection and Processing: Progress and Challenges in Biodiverse Reference Genome Creation

Version: Original Submission **Date:** 12/3/2024

Reviewer name: Robert Murphy

Reviewer Comments to Author:

Review of Lawniczak et al. Best Practice Guidance for Earth BioGenome Project Sample Collection and Processing: Progress and Challenges in Biodiverse Reference Genome Creation.The important manuscript is very well-written and organized, and the authors are to be complemented on this. The manuscript offers many links to relevant websites, and in doing so it is a valuable resource.The manuscript is particularly applicable to family-level collecting, but will have limited utility in the long term. For example, once the project moves to genus-level analyses, specimen acquisition will increasingly require extended fieldwork in remote areas. This activity will complicate the initial freezing of samples on dry ice as return to a laboratory or place where LN2 or dry ice is easily acquitted will be impossible. Although this is mentioned, I would suggest that the authors might want to add a bit of text relative to this eventuality.I am surprised that the manuscript does not mention CITES other than "It is also important that permissions are obtained for… shipping …" Shipping of CITES I species is quite complicated in requiring both export and import permits. One means to facilitate such shipments involves using CITES Exempt Permits held by museum, and rarely other institutions. Perhaps the authors should expand a bit on shipping constraints. Another issue, of course, is that some countries to not allow the exportation of bioresources (e.g., India).Re. "Researchers are encouraged to discuss directly with the NCBI Taxonomy curators (or the curators at ENA) [note space typo] whenever there is an opportunity to improve the NCBI Taxonomy, including taxonomic revisions and reconciliations." Unfortunately, for species identifications, changes can only be made by the submitter of the data, and once an error is created, publications usually perpetuate it. Thus, I believe the text might be better-phrased to reflect current practices at NCBI.Re. "It is also worth bearing in mind that if you are sharing material with another institute and an MTA (material transfer agreement) is in place, make sure that the MTA states that remaining material can be shared with a third party if you so wish." I disagree with this as the voucher-holder must be the sole controller of resource distribution. There's an infamous case of looking at genetic variability in a species based on a few samples. No variability was found. As it turned out, all independent sequencing was done using the same sample, which had been distributed to several labs independently. At least all sequencing of the same individual was repeatable!

Level of Interest

Please indicate how interesting you found the manuscript: Choose an item.

Quality of Written English

Please indicate the quality of language in the manuscript: Choose an item.

Declaration of Competing Interests

Please complete a declaration of competing interests, considering the following questions:

- Have you in the past five years received reimbursements, fees, funding, or salary from an organisation that may in any way gain or lose financially from the publication of this manuscript, either now or in the future?
- Do you hold any stocks or shares in an organisation that may in any way gain or lose financially from the publication of this manuscript, either now or in the future?
- Do you hold or are you currently applying for any patents relating to the content of the manuscript?
- Have you received reimbursements, fees, funding, or salary from an organization that holds or has applied for patents relating to the content of the manuscript?
- Do you have any other financial competing interests?
- Do you have any non-financial competing interests in relation to this paper?

If you can answer no to all of the above, write 'I declare that I have no competing interests' below. If your reply is yes to any, please give details below.

'I declare that I have no competing interests

I agree to the open peer review policy of the journal. I understand that my name will be included on my report to the authors and, if the manuscript is accepted for publication, my named report including any attachments I upload will be posted on the website along with the authors' responses. I agree for my report to be made available under an Open Access Creative Commons CC-BY license (http://creativecommons.org/licenses/by/4.0/). I understand that any comments which I do not wish to be included in my named report can be included as confidential comments to the editors, which will not be published.

Choose an item.

To further support our reviewers, we have joined with Publons, where you can gain additional credit to further highlight your hard work (see: https://publons.com/journal/530/gigascience). On publication of this paper, your review will be automatically added to Publons, you can then choose whether or not to claim your Publons credit. I understand this statement.

Yes Choose an item.
