# Supplementary material for: Best-practice guidance for Earth BioGenome Project sample collection and processing: progress and challenges in biodiverse reference genome creation
Source: Gigascience. 2025 May 29;14:giaf041. doi: 10.1093/gigascience/giaf041 (PMC12121479; doi:10.1093/gigascience/giaf041)
Supplement: giaf041_Reviewer_2_Report_original_submission — Oliver A. Ryder -- 12/9/2024 [file giaf041_reviewer_2_report_original_submission.pdf]

Reviewer Report

Title: Best Practice Guidance for Earth BioGenome Project Sample Collection and Processing: Progress and Challenges in Biodiverse Reference Genome Creation

Version: Original Submission **Date:** 12/9/2024

Reviewer name: Oliver A. Ryder

Reviewer Comments to Author:

This manuscript is an effort to "outline recommended standards and considerations in sample acquisition and processing" for the Earth Biogenome Project (EBP).The paper's sections include background, recommendations for sampling, openly collating genome sequencing proposals and efforts underway, ethical collecting, confident species identification, robust and complete metadata, vouchering, selection and processing of samples, establishment of a community io for protocols, fostering inclusive collaboration, and consideration and recommendation of future modifications or improvements relevant to sample acquisition and processing.Addressing crucial aspects of the EBP involves consideration of diverse disciplines and technologies, requiring informed input from multiple authors. It appears that the sections of the paper may have been distributed to specific authors, this deduced from the variation in style and voice of the sections.All the sections address significant aspects of a comprehensive presentation and discussion of collection and processing of useful samples for generation of annotated reference genome assemblies across the Tree of Life. However, they are uneven in their scope and depth.A major revision of the manuscript can re-organize the inclusion of topics so that they appear together in logical order. The depth of the sections could be more uniform. Each section could state which constituents of the broad EBP community the section addresses, and if all, so stated. It will be helpful to make it as much like a manual as feasible. Reference to common elements among and between sections would also be helpful. For example, discussions of vouchering appear in several sections and are at times contradictory, i.e., vouchers or not; taxonomic identification or not. In some sections the tone is directive ("must") and in places is less so ("recommended"). For an international, diverse audience the tone of the prescriptions should be carefully explained and rationalized.Overall, the treatment of the ethical issues is less than what should be expected. The ultimate success of the EBP will be strongly influenced by perceptions across cultures, tribes and nationalities, and the signatories to the Convention on Biological Diversity (CBD). In the section on ethical collecting the Kunming-Montreal Global Biodiversity Framework (KMGBF) is not mentioned. Similarly,l the adherence to the CARE and FAIR principles and prior informed consent is not in the manuscript.In the discussion of Confident Species Identification, standards are called for that later in the manuscript are discussed as difficult to impossible.In the section of Robust and Complete Metadata, there are many links in the text to complex documents, the portion of which is not clearly explained. To better make the desired points, some summary or explanation of what is desired is warranted. References to identifiers appear in this and other sections. Given the importance, perhaps a separate section about this that clearly compiles and/or references relationships among databases and sample IDs. The discussion of a robust mechanism for updating the names and identifiers brings a wealth of

terms: taxids, taxonomies, ToL IDs, ENA or NCBI. Taxonomy query services, are discussed as well as timelines for reconciliation/revision. Why is the date for amendments suggested to be 6 months?In the section on Vouchering, recommended is that vouchers be deposited in publicly accessible collection facilities, such as natural history museums or herbaria and specifically in GGBN-member institutions. These are not uniformly available facilities in countries, thereby raising the issue of export/import, access and benefit sharing, and other issues addressed in the Kunming-Montreal Global Biodiversity Framework (KMGBF). The recommendation that high-quality images should accompany each contributed specimen, with documentation including all relevant morphological axes, similarly poses challenges to inclusion of all CBD signatories. The recommended equipment/workstations is expensive equipment, not uniformly available in all countries, raising the issue of export/import, Nagoya Protocol, benefit sharing, and KMGBF.In the section on Sample to Sequence, within the discussion of Specimen and Tissue Selection, the discussion of challenges and approaches to obtaining HMW DNA across taxa identifies the need for community discussion, data sharing, and need to undertake complex and redundant sampling and storage procedures, noting that "recommendations will vary." In the section on Specimen Processing, a SPECIMEN_ID is recommended, along with a photo of the sample tubes, voucher tag, and specimen.Here it might be useful to allude to in the discussion of identifiers as part of the chain linking data to specimen and harmonize the recommendations. The section on Sequencing the very smalldiscusses the array of challenges that must be addressed with species of minute mass/volume and provides suggestions, while alluding to uncertainties, again raising the issue of taxonomic identification and complete destruction of the individual in order to obtain a suitable specimen. Later, the use of BARCODES, is raised and it might well appear here.In the section on Cold chain challenges, tthe issue of transfer of specimens is brought up. This has been mentioned in other sections, but is an issue of significance, if transfer is across international borders, due to Nagoya and KMGBF. For this article to be useful into the future, this aspect needs more prominence and careful consideration for the success of the EBP.The proposal for a EBP protocols.io community is a very useful suggestion, allowing for a community participation in rapid updating and communicating significant information crucial to the success of the EBP (including the EBP anecdotes).The section on Fostering Inclusive Collaboration for Enhanced Impact seems to me to be first principle and therefore brought in earlier in the manuscript. The EBP will fail if is not or is perceived not to be inclusive, equitable, involve all signatories to the CBD, and incorporate the CARE and FAIR principles.In the section on Looking to the Future, the utility of DNA barcoding of libraries to assist in specimen and sample verification is brought up. An important point and that should be brought up in the earlier discussions of samples and taxids.This section ends with speculation of the crucial significance of AI in the EBP at all stages. This is warranted, but as it is expected to be impactful in ways. not currently evident, the ending of the manuscript in this way could be strengthened by reinforcing the value to human society and planetary ecosystems that support it, and of which humankind is a part. Having reference genomes across the Tree of Life, enabled by multiple technologies, including AI "contribute[s] to a deeperunderstanding of biodiversity for applications in conservation, ecology, and evolutionary biology."Additional comments are made on the attached PDF copy of the manuscript.

Level of Interest

Please indicate how interesting you found the manuscript: Choose an item.

Quality of Written English

Please indicate the quality of language in the manuscript: Choose an item.

Declaration of Competing Interests

Please complete a declaration of competing interests, considering the following questions:

- Have you in the past five years received reimbursements, fees, funding, or salary from an organisation that may in any way gain or lose financially from the publication of this manuscript, either now or in the future?
- Do you hold any stocks or shares in an organisation that may in any way gain or lose financially from the publication of this manuscript, either now or in the future?
- Do you hold or are you currently applying for any patents relating to the content of the manuscript?
- Have you received reimbursements, fees, funding, or salary from an organization that holds or has applied for patents relating to the content of the manuscript?
- Do you have any other financial competing interests?
- Do you have any non-financial competing interests in relation to this paper?

If you can answer no to all of the above, write 'I declare that I have no competing interests' below. If your reply is yes to any, please give details below.

I declare I have no competing interests.

I agree to the open peer review policy of the journal. I understand that my name will be included on my report to the authors and, if the manuscript is accepted for publication, my named report including any attachments I upload will be posted on the website along with the authors' responses. I agree for my report to be made available under an Open Access Creative Commons CC-BY license (http://creativecommons.org/licenses/by/4.0/). I understand that any comments which I do not wish to be included in my named report can be included as confidential comments to the editors, which will not be published.

Choose an item.

To further support our reviewers, we have joined with Publons, where you can gain additional credit to further highlight your hard work (see: https://publons.com/journal/530/gigascience). On publication of this paper, your review will be automatically added to Publons, you can then choose whether or not to claim your Publons credit. I understand this statement.

Yes Choose an item.
